# Human Non-neutralizing HIV-1 Envelope Monoclonal Antibodies Limit the Number of Founder Viruses during SHIV Mucosal Infection in Rhesus Macaques

**DOI:** 10.1371/journal.ppat.1005042

**Published:** 2015-08-03

**Authors:** Sampa Santra, Georgia D. Tomaras, Ranjit Warrier, Nathan I. Nicely, Hua-Xin Liao, Justin Pollara, Pinghuang Liu, S. Munir Alam, Ruijun Zhang, Sarah L. Cocklin, Xiaoying Shen, Ryan Duffy, Shi-Mao Xia, Robert J. Schutte, Charles W. Pemble IV, S. Moses Dennison, Hui Li, Andrew Chao, Kora Vidnovic, Abbey Evans, Katja Klein, Amit Kumar, James Robinson, Gary Landucci, Donald N. Forthal, David C. Montefiori, Jaranit Kaewkungwal, Sorachai Nitayaphan, Punnee Pitisuttithum, Supachai Rerks-Ngarm, Merlin L. Robb, Nelson L. Michael, Jerome H. Kim, Kelly A. Soderberg, Elena E. Giorgi, Lily Blair, Bette T. Korber, Christiane Moog, Robin J. Shattock, Norman L. Letvin, Joern E. Schmitz, M. A. Moody, Feng Gao, Guido Ferrari, George M. Shaw, Barton F. Haynes

**Affiliations:** 1 Center for Virology and Vaccine Research, Beth Israel Deaconess Medical Center, Harvard Medical School, Boston, Massachusetts, United States of America; 2 Duke Human Vaccine Institute, Duke School of Medicine, Durham, North Carolina, United States of America; 3 Department of Medicine, Perelman School of Medicine, University of Pennsylvania, Philadelphia, Pennsylvania, United States of America; 4 Department of Medicine, St Mary’s Campus, Imperial College London, London, United Kingdom; 5 Department of Pediatrics, Tulane University School of Medicine, New Orleans, Louisiana, United States of America; 6 Division of Infectious Diseases, Department of Medicine, University of California, Irvine, Irvine, California, United States of America; 7 Tropical Hygiene, Mahidol University, Bangkok, Thailand; 8 Armed Forces Research Institute of Medical Sciences (AFRIMS), Bangkok, Thailand; 9 Clinical Tropical Medicine, Mahidol University, Bangkok, Thailand; 10 Department of Disease Control, Ministry of Public Health, Nonthaburi, Thailand; 11 US Military Research Program, Walter Reed Army Institute of Research, Silver Spring, Maryland, United States of America; 12 Theoretical Division, Los Alamos National Laboratory, Los Alamos, New Mexico, United States of America; 13 U1109, INSERM University of Strasbourg, Strasbourg, Alsace, France; Vaccine Research Center, UNITED STATES

## Abstract

HIV-1 mucosal transmission begins with virus or virus-infected cells moving through mucus across mucosal epithelium to infect CD4^+^ T cells. Although broadly neutralizing antibodies (bnAbs) are the type of HIV-1 antibodies that are most likely protective, they are not induced with current vaccine candidates. In contrast, antibodies that do not neutralize primary HIV-1 strains in the TZM-bl infection assay are readily induced by current vaccine candidates and have also been implicated as secondary correlates of decreased HIV-1 risk in the RV144 vaccine efficacy trial. Here, we have studied the capacity of anti-Env monoclonal antibodies (mAbs) against either the immunodominant region of gp41 (7B2 IgG1), the first constant region of gp120 (A32 IgG1), or the third variable loop (V3) of gp120 (CH22 IgG1) to modulate *in vivo* rectal mucosal transmission of a high-dose simian-human immunodeficiency virus (SHIV-BaL) in rhesus macaques. 7B2 IgG1 or A32 IgG1, each containing mutations to enhance Fc function, was administered passively to rhesus macaques but afforded no protection against productive clinical infection while the positive control antibody CH22 IgG1 prevented infection in 4 of 6 animals. Enumeration of transmitted/founder (T/F) viruses revealed that passive infusion of each of the three antibodies significantly reduced the number of T/F genomes. Thus, some antibodies that bind HIV-1 Env but fail to neutralize virus in traditional neutralization assays may limit the number of T/F viruses involved in transmission without leading to enhancement of viral infection. For one of these mAbs, gp41 mAb 7B2, we provide the first co-crystal structure in complex with a common cyclical loop motif demonstrated to be critical for infection by other retroviruses.

## Author Summary

Antibodies specifically recognize antigenic sites on pathogens and can mediate multiple antiviral functions through engagement of effector cells via their Fc region. Current HIV-1 vaccine candidates induce polyclonal antibody responses with multiple antiviral functions, but do not induce broadly neutralizing antibodies. An improved understanding of whether certain types of non-neutralizing HIV-1 specific antibodies can individually protect against HIV-1 infection may facilitate vaccine development. Here, we test whether non-neutralizing antibodies with multiple antiviral functions mediated through FcR engagement and recognition of virus particles or virus-infected cells can limit infection, despite lacking classical virus neutralization activity. In a passive antibody infusion-rhesus macaque challenge model, we tested the ability of non-neutralizing monoclonal antibodies to limit virus acquisition. We demonstrate that two different types of non-neutralizing antibodies, one that recognizes both virus particles and infected cells (7B2) and another that recognizes only infected cells (A32) were capable of decreasing the number of transmitted founder viruses. Further, we provide the structure of 7B2 in complex with the gp41 cyclical loop motif, a motif critical for entry. These findings provide insights into the role that antibodies with antiviral properties, including virion capture and FcR mediated effector function, may play in protecting against HIV-1 acquisition.

## Introduction

The induction of HIV-1 broadly reactive neutralizing antibodies (bnAbs) by experimental vaccines is a critical goal of HIV-1 vaccine development efforts. However, bnAbs cannot be induced by existing HIV-1 vaccine candidates [1]. The RV144 ALVAC/AIDSVAX B/E HIV-1 vaccine efficacy trial demonstrated 31.2% estimated vaccine efficacy 42 months after the vaccination regimen was initiated [2]. Antibodies that mediated antibody dependent cell-mediated cytotoxicity (ADCC), or Tier 1 neutralizing antibodies in the presence of low envelope IgA antibodies, were identified as correlates of decreased transmission risk [3–6]. Thus, there is considerable interest in determining if commonly elicited ADCC-mediating, but non-broadly neutralizing antibodies against HIV-1 envelope have potential for protection against transmission [7,8].

Holl *et al*. have described non-neutralizing antibodies that bind to the immunodominant region (aa 579–613) of gp41 that can prevent HIV-1 infection of macrophages *in vitro* [9]. Others have demonstrated that these types of gp41 immunodominant antibodies bind to virions [10–12] and mediate ADCC [13,14]. Recently the HIV-1 gp41 immunodominant loop structure was determined for the first time in the context of the pre-fusion viral spike [15]. In that structure, the loop was disulfide bonded and buried under the trimer gp120 head groups and other elements of the observed pre-fusion gp41 fold [15]. However, to date, the only antibody to the immunodominant loop with its structure determined is that of unliganded mAb 3D6 [16,17].

Ferrari *et al*. [18], Guan *et al*. [19], Bonsignori *et al*. [20] and Veillette *et al*. [21,22] have described non-neutralizing gp120 antibodies that bind to a conformational epitope on Env in the first constant (C1) region of primary virus-infected CD4^+^ T cells were potent mediators of ADCC. The crystal structures of the C1 conformational A32-like antibodies N5-i5 and 2.2c were found to recognize overlapping epitopes formed by mobile layers 1 and 2 of the gp120 inner domain, including the C1 and C2 regions, but bind gp120 at different angles via juxtaposed VH and VL contact surfaces [23].

Mucosal transmission involves a series of events wherein HIV-1 or HIV-1-infected cells traverse genital tract mucus and epithelia, and subsequently infect epithelial or sub-mucosal CD4^+^ T cells [24,25]. Theoretically, antibodies that can bind virions at the initial stages of the transmission event [26] might be able to prevent virus movement across mucus and epithelium, thus preventing infection. Moreover, antibodies that mediate ADCC may be able to sensitize infected CD4^+^ T cells in the submucosa for natural killer (NK) cell killing [18,19], and abort transmission events. Alternatively, some studies have raised the issue that non-neutralizing antibodies might enhance infection [27–30].

BnAbs passively administered to rhesus macaques provide protection from SHIV challenges at mucosal surfaces [31]. Fc receptor interactions with natural killer (NK) cells are important for CD4 binding site bnAb neutralization in the macaque model [32]. However, non-neutralizing Env antibodies, when administered systemically or locally, have been reported to have only limited or no protective effect against vaginal SHIV challenge in rhesus macaques [30]. Moog *et al*. have demonstrated that local administration of gp41 immunodominant region non-neutralizing mAbs did not prevent infection with SHIV but did slow the onset of viremia in one of six animals and blunted the peak viremia in two others [13].

Enumeration of transmitted/founder (T/F) genomes has proved to be an important method of discerning the number of infecting viruses in various clinical settings [33,34], and has been used to monitor infection in macaques following SHIV challenge [30]. In this latter study, the mean number of T/F viruses was approximately 1 in most groups, thus, the contribution of non-neutralizing antibodies in limiting founder viruses compared to the control antibody was difficult to analyze [30]. Instead, this study showed a statistically significant enhancement in the number of founder viruses resulting in productive clinical infection in animals treated with the non-neutralizing CD4bs mAb, b6 [30]. Thus, the critical question remains as to whether antibodies that have an effector profile including recognition of virus particles and/or engaging FcR on effector cells can prevent HIV-1 transmission to any degree, or conversely, do such antibodies enhance infection?

In this study, we used T/F virus enumeration as a measure of relative protection from infection along with VL set point and CD4 preservation. We studied the activity of two human antibodies with characteristics of antibodies commonly induced by HIV-1 vaccine candidates, mAb 7B2 IgG1_AAA directed against the envelope gp41 immunodominant region [11,35] and the gp120 C1 mAb A32 IgG1_AAA, in the setting of high dose SHIV-BaL mucosal challenge. We show that while these non-neutralizing mAbs were unable to reduce the rate of productive infection in the high dose SHIV-BaL rhesus macaque challenge model, they reduced the number of T/F viruses involved in transmission events. Importantly, the challenge studies did not show evidence of antibody-mediated enhancement of virus infection.

## Results

### Characterization of mAb 7B2 epitope specificity

**Fig 1:**
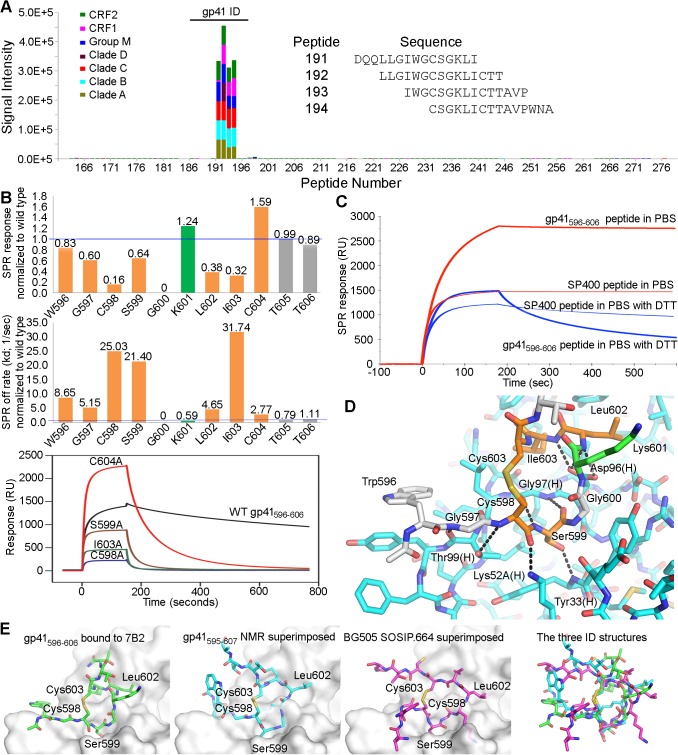
Fab and FcR binding. (**A**) Linear cross-clade epitope mapping of 7B2 IgG1_AAA by peptide microarray. FcR binding (response units), on-rate (ka) and off rate (kd) by Surface Plasmon Resonance (SPR) of 7B2 IgG1_AAA. (**B**) Fine mapping of the 7B2 epitope within the gp41 immunodominant loop. Top Graph shows the binding response at saturation (~140 seconds after starting injection of 7B2 Fab) of each Ala-substituted gp41_596-606_ peptide normalized to wild type and the middle graph shows the normalized off-rate of the same peptides. Data are representative of at least two measurements on adjacent spots in the same sensor chip. Residues that are part of the 7B2 epitope are colored in orange. The Lys601Ala mutant peptide is highlighted in green since it gave a higher binding response and a decreased off-rate. Bottom graph is an example of sensogram showing 7B2 Fab binding to WT and select Alanine mutant gp41_596-606_ peptides that were used to generate the top and middle graphs. (**C**) Binding between 7B2 and gp41 peptides in standard and reducing conditions. (**D**) The structure of the 7B2 Fab-gp41 peptide complex shows detailed polar interactions. Hydrogen bonds between functional groups in the peptide and the heavy chain of the Fab are indicated. (**E**) Comparison of the gp41 ID loop from our structure (far left) against its structure obtained from NMR (middle left) [38,39] and its conformation as shown in the BG505.SOSIP.664 structure (middle right) [15] superimposed against the 7B2 paratope. A superposition of all three ID conformations (far right) highlights the conformational variability of this region.

The epitope specificities of two of the three HIV-1 Env mAbs examined in the rhesus macaque challenge model (i.e. A32 IgG [18] and CH22 IgG [36]) have already been previously described. Thus, here we characterized the epitope specificity of 7B2 IgG1. 7B2 IgG1 mAb recognizes a linear epitope in the gp41 immunodominant region with cross-clade reactivity (clades A, B, C, D, CRF1 and CRF2) as measured by peptide microarray [37] (Fig 1A). Specific interacting residues within this gp41_596-606_ 11mer were examined with peptide alanine substituted mutants and surface plasmon resonance assays (Fig 1B). The footprint from epitope mapping demonstrated that residues in the 7B2 epitope were Cys598, Gly600, Leu602, Ile603, and Cys604. Ala substitution of Cys604 resulted in a peptide that bound with a higher peak response, yet had an off-rate approximately 2.8 times that of the Cys604 peptide. From these data we postulated that Cys604 mediated cyclization of the peptide that would be critical for the generation of a stable 7B2-peptide complex. Therefore we repeated the binding experiments of 7B2 using both longer and minimal epitope-containing wild-type gp41 peptides in standard (oxidizing) and reducing conditions (Fig 1C). In reducing conditions, the steady state binding of 7B2 mAb to both the longer and shorter peptides was at a relatively lower level with 100 to 150 fold increased off rates. These data suggested two primary factors for antibody binding, first, a disulfide-bonded, cyclical structure, and second, an induced fit is a likely factor in binding.

### 7B2 Fab crystal structure analysis

The co-crystal structure of 7B2 Fab bound to a gp41_596-606_ peptide was solved by molecular replacement and refined to a resolution of 2.7 Å (Table 1). The most prominent finding in the structure occurred in the central segment of the epitope-bearing peptide where a closed loop was displayed by virtue of the disulfide bond between Cys598 and Cys604. The cyclical conformation of the gp41 peptide was fostered by Gly600, where any side chain would diminish the backbone's ability to adopt the closed conformation and would clash with the side chain of Tyr32 in CDR-H1. Overall, the majority of the contacts between 7B2 antibody and the gp41 nominal peptide occurred through the base of CDR-H3 and the cyclical portion of the gp41 peptide (Figs 1D and S1). We concluded that the cysteine-cysteine bond and resulting loop conformation of the gp41 immunodominant region was indispensable for 7B2 binding.

**Table 1:** Data collection and refinement statistics for the 7B2 Fab-gp41 _596–606_ structure.

Data collection^a^	
**Space group:**	P2_1_ (P12_1_1)
***Cell dimensions***	
**a, b, c (Å):**	73.4, 76.3, 127.6
**α, β, γ (°):**	90.0, 98.1, 90.0
**Resolution (Å)** ^b^ **:**	50.0–2.7 (2.75–2.70)
**R**_**merge**_ **(%):**	3.7 (67.6)
**<I/σ>**	31.0 (1.6)
**Completeness (%):**	99.1 (98.7)
**Redundancy:**	4.9 (5.0)
**Refinement**	
**Total # reflections:**	35499 (2135)
**Unique # reflections:**	7245 (427)
**Rwork / Rfree (%)**	18.9/22.3 (32.9/34.5)
**Average B factor (Å** ^**2**^ **)**	99.2
**Nonhydrogen atoms**	6806
**Water molecules**	91
***R*.*M*.*S*. *deviations***	
**Bond lengths (Å):**	0.006
**Bond angles (°):**	0.992
**ψ, φ favored (%):**	97.3
**ψ, φ allowed (%):**	2.7
**ψ, φ outlying (%):**	0.0

^a^ The crystal had two Fab-peptide complexes in the asymmetric unit. The dataset came from a single crystal.

^b^ Values in parentheses are for the highest resolution shells.

The disulfide-linkage of the gp41 immunodominant loop in the 7B2 complex structure was similar to those seen in the chain reversal regions of other viruses [40–45]. However, the only component of the chain reversal motif that HIV-1 has in common with filoviruses and most retroviruses is the core, disulfide-linked motif, where it likely served the same function as in other retroviruses, specifically stabilization of the chain reversal region and a role in the transition state and formation of the 6HB [40,42,46].

The structure of the unliganded, wild-type gp41 immunodominant loop has been determined in solution by NMR [38,39], however a superposition of the solution structure upon that seen in our complex did not suggest a compatible conformation for strict docking (Fig 1E). Similar comparisons were seen between the coordinates of the gp41 peptide in our crystal structure and the coordinates of the SIV gp41 and HIV-1 gp41 solution structures [47,48]. The structure of the gp41 immunodominant loop has also been determined in the context of the Env pre-fusion trimer [15]. The immunodominant loop as seen in the BG505 SOSIP.664 structure resembled neither the conformation in the 7B2 complex structure nor the NMR solution peptide structures (Fig 1E). A radically different conformation of the polypeptide backbone atoms within the loop resulted in different positions for several key residues. The conformational variability of the immunodominant loop is evident in comparing these structures. Thus, we concluded that the wild-type 7B2 epitope-containing peptide was likely subject to induced fit though we could not rule out conformational selection of the disulfide-bonded structure as observed in other published cases of antibody-antigen recognition [49–53]. Moreover, the immunodominant loop appeared in an ordered and disulfide-bonded state in the SOSIP structure, but it was buried beneath glycoprotein. Thus, the immunodominant loop is inaccessible on pre-fusion spikes though it is present on Env stumps [54–59], so 7B2 and antibodies like it may capture virus and bind to virus-infected cells but fail to neutralize because they bind stumps and post-fusion structures.

### FcR binding

**Fig 2:**
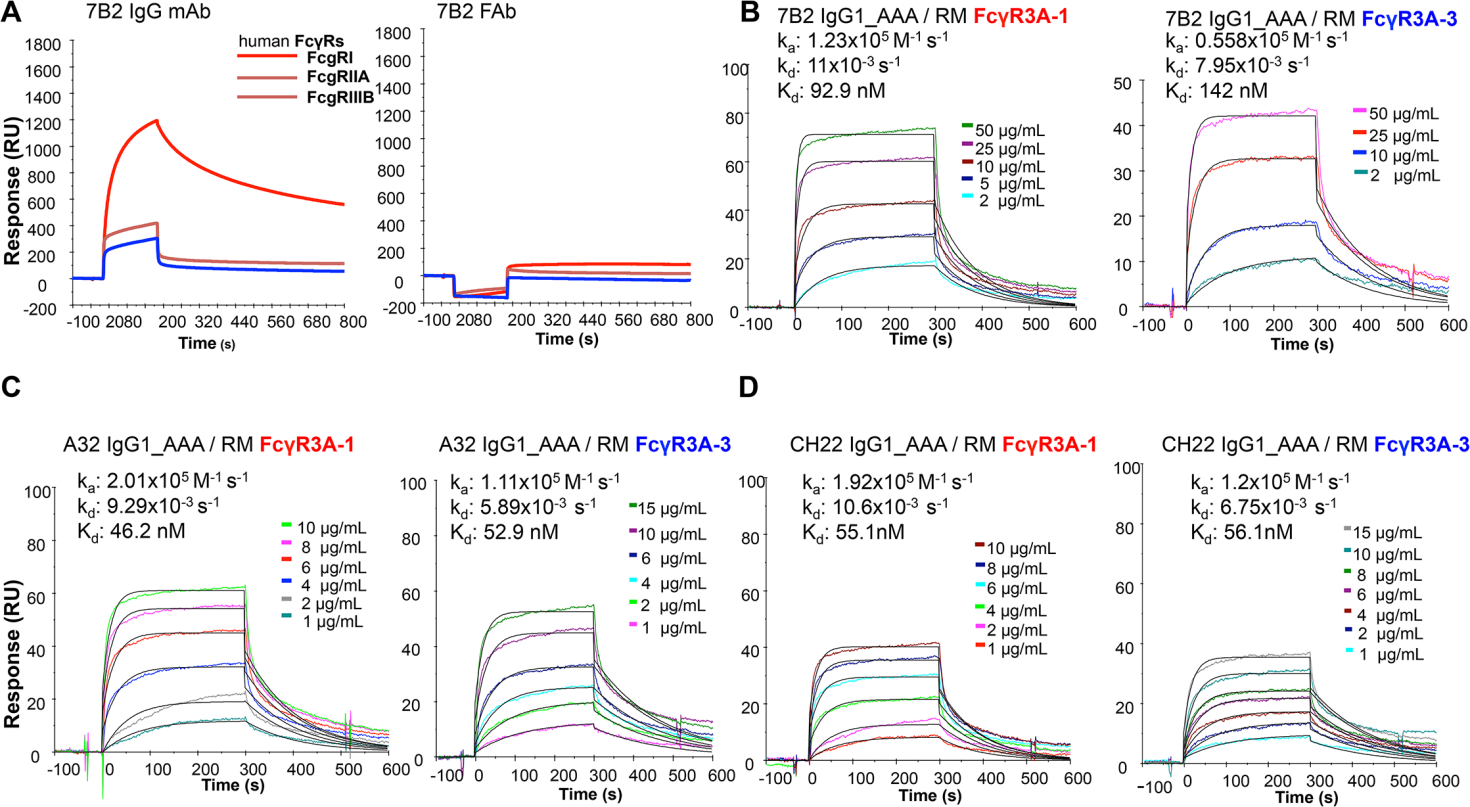
Surface plasmon resonance of mAbs to human and rhesus FcR. **(A)** 7B2 IgG1_AAA and Fab to human FcR FcgRI, FcgRIIA, FcgRIIIB and **(B)** rhesus macaque FcgR3A-1 and _FcgR3A-3. **(C)** A32 IgG1_AAA and **(D)** CH22 IgG1_AAA to rhesus macaque FcgR3A-1 and _FcgR3A-3.

We constructed 7B2 IgG1 optimized for binding to human FcRIII (CD16) by introducing alanine substitutions at positions 298, 333 and 334 (S298A, E333A and K334A) [60]. The ability of 7B2 IgG1_AAA to mediate antiviral function depends on both antigen recognition as well as engagement of FcγR on effector cells. We determined the binding of mAb 7B2 IgG_AAA to human FcγR1 (CD64), FcγRII (CD32) and FcγRIIIa (CD16) by SPR measurements (Fig 2A). 7B2 IgG1_AAA mAb bound with high affinity to FcγRI/CD64 (K_d_ = 49 nM) and with lower affinities to FcγRII/CD32a (K_d_ = 0.17 μM) and FcγRIIIa/CD16 (K_d_ = 1.1 μM) proteins. Among the three FcγR proteins, binding to FcγRIIIa (CD16) displayed fast kinetics, with an off rate that was almost two orders of magnitude faster when compared to FcγRI binding. The range of the observed differences in binding Kd and kinetics of 7B2 IgG_AAA mAb was consistent with previous report of IgG binding to the three classes of human Fc receptors [61–63]. As expected, the 7B2 IgG1_AAA Fab fragment control did not bind any FcRs. (Fig 2A). We next tested the binding of 7B2 IgG1 mAb to rhesus FcR (FcγRIIIa-1/-2 and FcγRIIIa-3). 7B2 IgG1mAb bound most avidly to rhesus macaque FcγR3A (Fig 2B). 7B2_AAA bound to rhesus FcγR3A (both allelic variants) with higher affinities (due to slower off-rates) than 7B2_SEK, which contains Fc region aa S298, E333 and K334 (Table 2). CH22 and A32 mAbs bound to both human FcγR3A-1 and FcγR3A-3 with similar affinities (Fig 2C and 2D, Table 2).

**Table 2:** Binding rate constants and affinities of rhesus FcγR3 binding to mAbs.

mAb	Measurement	FcγR3A-1	FcγR3A
**7B2 IgG1_SEK**	**k**_**a**_ **(x 10** ^**5**^ **M** ^**-1**^ **s** ^**-1**^ **)**	1.6+/- 0.8	0.46+/- 0.1
	**k**_**d**_ **(x 10** ^**−3**^ **s** ^**-1**^ **)**	39.6+/- 16.6	23.9+/- 4.3
	**K**_**d**_ **(nM)**	267.7+/- 40.5	534.7+/- 56.6
**7B2 IgG1_AAA**	**k**_**a**_ **(x 10** ^**5**^ **M** ^**-1**^ **s** ^**-1**^ **)**	1.7+/- 0.5	0.65+/- 0.1
	**k**_**d**_ **(x 10** ^**−3**^ **s** ^**-1**^ **)**	10.3+/- 0.6	9.4+/- 1.3
	**K**_**d**_ **(nM)**	68.7+/- 21.5	147.3+/- 19.6
**A32 IgG1_ AAA**	**k**_**a**_ **(x 10** ^**5**^ **M** ^**-1**^ **s** ^**-1**^ **)**	1.9+/- 0.08	1.1
	**k**_**d**_ **(x 10** ^**−3**^ **s** ^**-1**^ **)**	9.4+/- 0.6	6.1
	**K**_**d**_ **(nM)**	50.0+/- 5.0	56.0
**CH22 IgG1_AAA**	**k**_**a**_ **(x 10** ^**5**^ **M** ^**-1**^ **s** ^**-1**^ **)**	1.7	1.2
	**k**_**d**_ **(x 10** ^**−3**^ **s** ^**-1**^ **)**	8.4	5.6
	**K**_**d**_ **(nM)**	47.8	47.9

Antibodies were measured for rhesus FcR binding by surface plasmon resonance (SPR). Mean and standard deviation from 3 independent assays are indicated where available.

To confirm that 7B2 IgG1_AAA Fc bound to rhesus FcRs on monocytes, we coated the SP400 gp41 immunodominant region peptide on sheep red blood cells (SRBC), then coated the SP400- SRBC with 7B2 IgG1_AAA or a negative control antibody. Next we compared the abilities of human and rhesus monocytes to phagocytose the 7B2 IgG1_AAA-opsonized SRBC. Rhesus monocytes were able to phagocytose 7B2 IgG1_AAA mAb-coated SRBC equally as well as human monocytes (55 +/- 10% of rhesus monocytes with ≥2 internalized SRBC; 57 +/- 7% of human monocytes with ≥2 internalized SRBC).

### MAb 7B2 IgG1_AAA captured HIV-1 and SHIV virions

**Fig 3:**
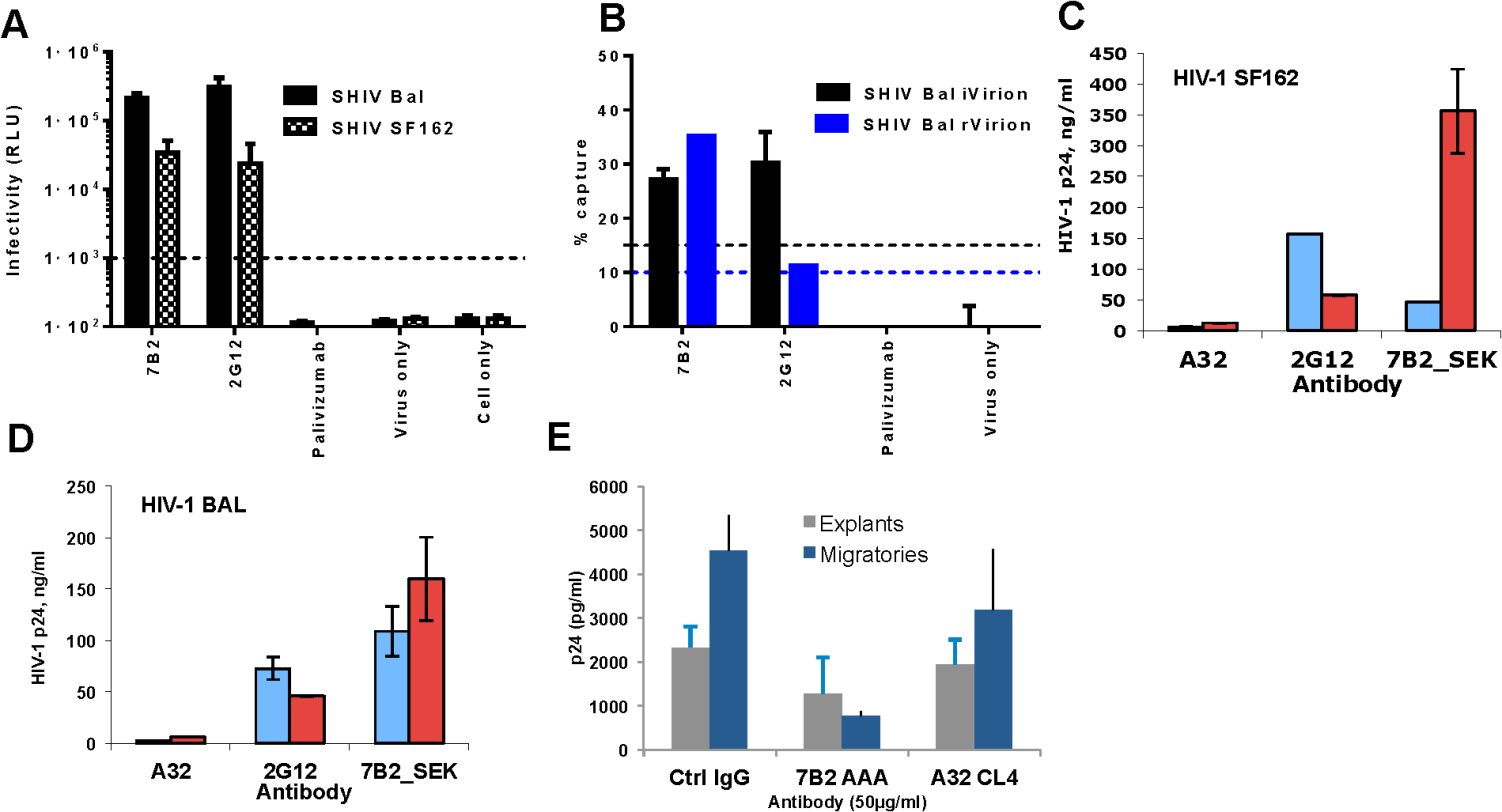
7B2 mAb captures infectious SHIV BaL and SF162. SHIV BaL and SHIV SF162 virus capture by 7B2 and controls were measured by either **(A)** a plate–based capture assay (relative luciferase unit (RLU) on day 7 post infection shown) **(B)** or a column-based assay (% virus capture based on SIV *gag* viral RNA measurement for rVirion and RLU infectivity for iVirion percentages, respectively. The error bar is the SEM of three wells replicates. The dashed line is the positivity cutoff. The level of virion capture in the presence (red bars) and absence (blue bars) of soluble CD4 for HIV-1 SF162 **(C)** and HIV- BAL **(D)** are shown. Non-neutralizing mAb A32 and a neutralizing mAb 2G12 were used as negative and positive controls, respectively. Error bars show mean ± SEM from 3 separate experiments. **(E)** 7B2_AAA does not inhibit infection of rectal explants (gray) but does inhibit infectious transfer from migratory cells that emigrate from mucosal tissue (at the highest concentration (50 μg) (blue). This is reflective of CD4+ T cells being the primary targets of infection. Results are the average of two experiments.

It was important to determine if the 7B2 IgG1_AAA mAb could capture infectious HIV-1 virions using an assay that could differentiate between infectious and noninfectious virus using both viral RNA and an infectious virus readout [11,64]. We previously reported that whereas mAb 2G12 captured the majority of infectious virus, mAb 7B2 IgG1_AAA captured both non-infectious as well as infectious virions [11,12,64]. However, mAb 7B2 IgG1_AAA could not, in any experiment, capture all infectious virions. Thus, mAb 7B2 IgG1_AAA captured a subset of both infectious and non-infectious virus particles. Here we tested whether mAb 7B2 IgG1_AAA could capture the infectious CCR5- tropic SHIV virions (SHIV-BaL and SHIV-SF162P3). As with HIV-1, 7B2 IgG1_AAA was able to capture a portion of infectious virions of both SHIVs (Fig 3A and 3B). In contrast, A32 mAb did not significantly capture any virions (Fig 3C and 3D).

We next asked whether recognition of HIV-1 virions by 7B2 IgG1_AAA mAb was dependent on CD4 binding. Using a virion capture assay that measures capture of virus particles without an infectious readout [65] we determined whether sCD4 binding to virus makes it more susceptible to capture by 7B2 IgG1_AAA mAb. We found that mAb 7B2 IgG1_AAA capture of HIV-1 SF162.B, BG1168.B, 6535.B, 6846.B and CAP 45.C virions, was augmented in the presence of sCD4 (S1 Table). Similarly, mAb 7B2 IgG1_AAA effectively captured parental HIV-1 SF162.B and BaL.B as well as SHIV-SF162P3 and SHIV-BaL (Fig 3C and 3D) in the presence of sCD4.

### 7B2 IgG1_AAA does not inhibit HIV-1 infection of rectal explant cultures but reduces dendritic cell dissemination of virus

The ability of 7B2 IgG1_AAA mAb to prevent infection of human colorectal tissue was assessed using an established ex-vivo rectal explant model [66]. 7B2 IgG1_AAA mAb had no direct impact on infection of colorectal explants (Fig 3E). 7B2 IgG_AAA IgG1 mAb did reduce dissemination of infection from dendritic cells that emigrate from the tissue during the first 24 hours of culture on incubation with CD4+ indicator T cells (Fig 3E). However, 7B2 and A32 IgG1 mAbs did not inhibit infection of monocyte derived DC or DC mediated trans-infection of co-culture with T cells (S2 Fig).

### 7B2 IgG_AAA neutralization of HIV-1 in blood monocytes and peritoneal macrophages

**Fig 4:**
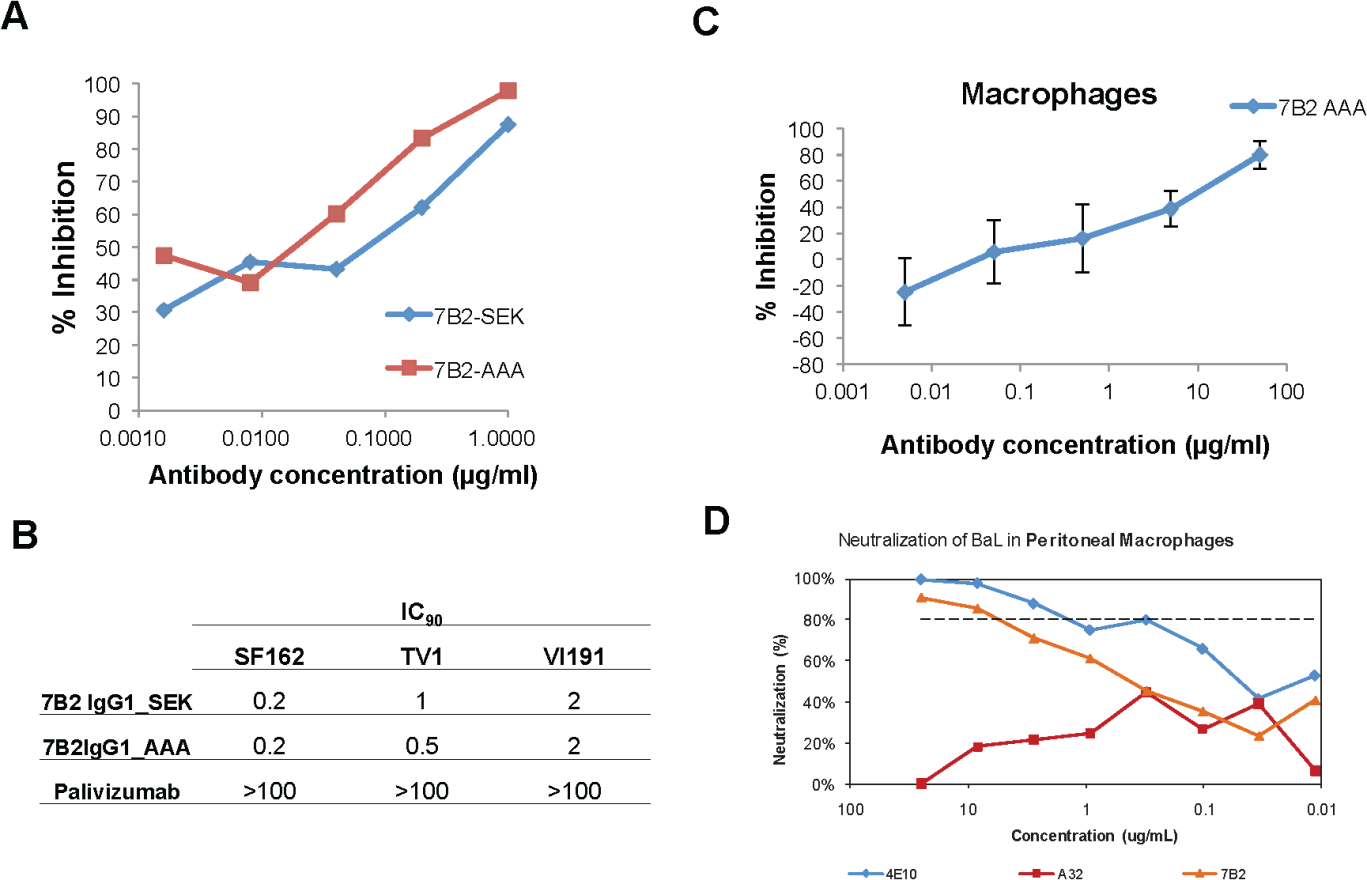
Macrophage neutralization assays. (**A)** HIV-1 Bal infected macrophages are inhibited in a dose dependent manner by 7B2-SEK and 7B2-AAA mAbs. Palivizumab did not neutralize (>100 IC_90_). (**B**) 7B2 mAb neutralization of HIV-1 SF162 (Subtype B), HIV-1 TV-1 (Subtype C), and HIV-1 Vl191. (**C**) 7B2_AAA displays a dose dependent inhibition of HIV infection of monocyte-derived macrophages. (**D)** Neutralization of BaL in peritoneal macrophages. gp41 Env specific Ab (7B2 mAb) neutralizes HIV- infection in human peritoneal macrophages. Virus input was normalized to RNA copies/mL. HIV replication was quantified by measuring the amount of luciferase in macrophage lysates.

MAb 7B2 IgG1_AAA when expressed in both the SEK and AAA IgG1 backbones effectively neutralized HIV-1 BaL with ID_50_s of 0.05 μg/ml in peripheral blood monocytes differentiated into macrophages (Fig 4A). The negative control, anti-respiratory syncytial virus mAb, palivizumab, had no inhibitory effect at 100 μg/mL. 7B2 IgG1 mAb also neutralized SF162 (0.2 IC90 for both the SEK and _AAA mAbs) and TV-1 (1 IC_90_ for SEK and 0.5 for _AAA) (Fig 4B). In another assay, 7B2 IgG1 mAb was confirmed to mediate virus inhibition of infection in macrophages [9] (Fig 4C). The negative control IgG and A32 mAb at 50 μg/ml did not block infection (107%, 109% of control infection), respectively. Moreover, 7B2 IgG1 mAb could mediate virus inhibition in tissue derived peritoneal macrophages (Fig 4D) and in antibody-dependent cell mediated virus inhibition assays (ADCVI) (S2 Fig).

### Antibody dependent cellular cytotoxicity (ADCC)

**Fig 5:**
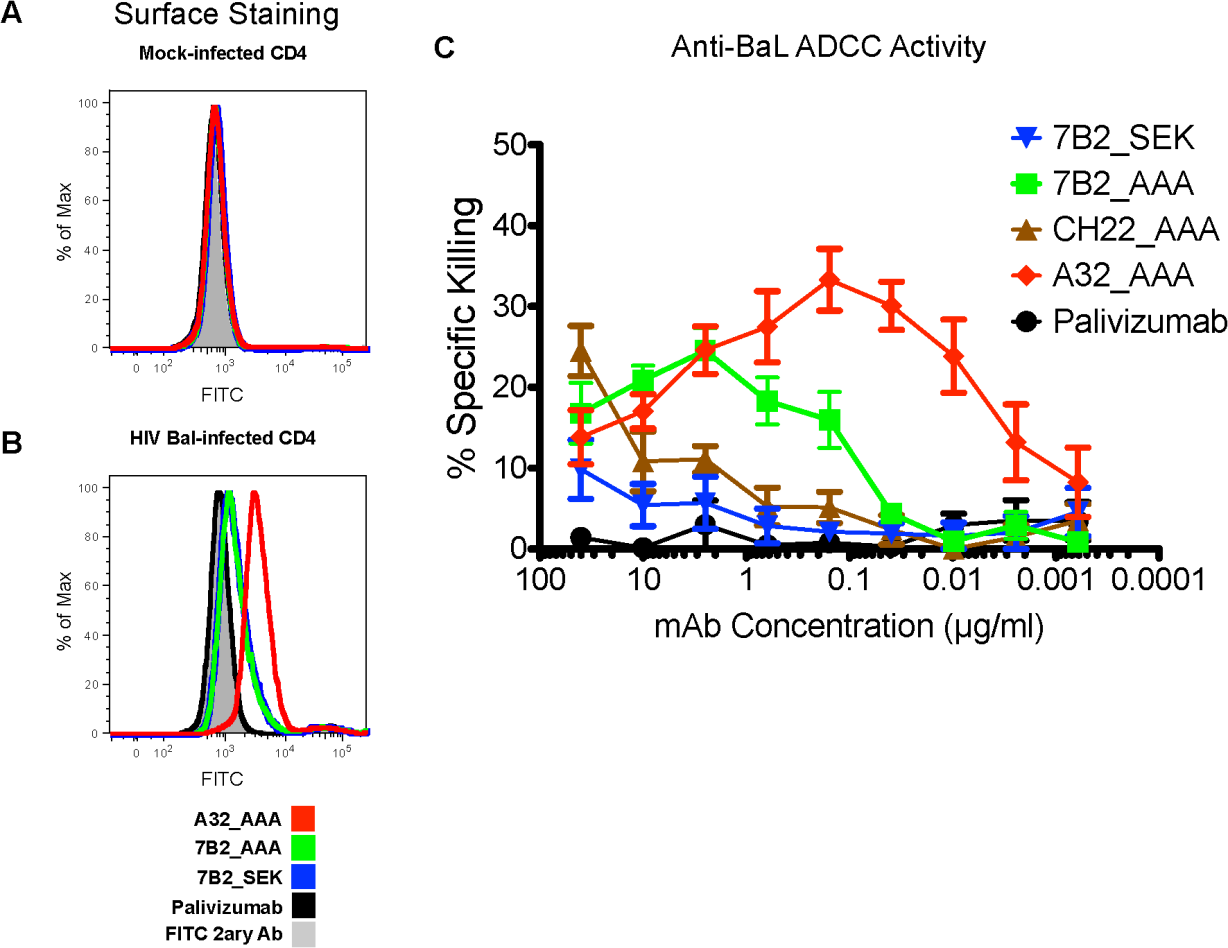
Ability of HIV-1 Env-specific mAbs to bind HIV-1 infected cells and mediate ADCC. **(A)** Mock infected primary human CD4+ T cells and (**B)** HIV-1 IMC_BaL_ infected primary human CD4+ T cells were incubated with the indicated mAbs, and binding was detected by secondary staining with a FITC-conjugated anti-human IgG antibody. (**C**) ADCC activities of A32, CH22, and 7B2 mAbs against HIV-1IMC_BaL_-infected CEM.NKR_CCR5_ CD4^+^ targets cells in the presence of NK cells. Results are the average of three experiments +/- SEM.

To determine if 7B2 IgG1_AAA mAbs could coat virus infected targets and arm natural killer (NK) cell effectors for antibody dependent cellular cytotoxicity (ADCC), we first confirmed the ability of 7B2 IgG1 SEK, 7B2 IgG1_AAA and A32 IgG1 mAb to bind to the surface of HIV-1 B.BaL infected CD4+ T cells (Fig 5A and 5B). Palivizumab and A32_AAA were included as negative and positive controls, respectively. Both the SEK and _AAA versions of mAb 7B2 IgG1 had similar ability to bind the surface of HIV-1 B.BaL infected cells. Since our challenge stock was SHIV-BaL, we next assayed for the ability of mAb 7B2 IgG1_SEK (and 7B2 IgG1_AAA to mediate ADCC against HIV-1 B.BaL-infected CD4 T cells. We found that 7B2 IgG1_AAA as well as A32_AAA IgG1 mAb, and the neutralizing V3-region specific mAb CH22 IgG1_AAA could indeed mediate killing of HIV-1 B.BaL-infected CD4+ T cells. In contrast, when mutation of the S298A as well as E333A and K334A in the AAA form was reverted back to SEK in the Fc domain of the mAb, it reduced the ability of the 7B2 IgG1 mAb to mediate ADCC (Fig 5C). Therefore we used the 7B2 IgG1_ AAA mutant for passive infusion into macaques to determine the ability to protect against SHIV-BaL intrarectal challenge.

### 7B2 mAb, A32 mAb and CH22 mAb binding to rhesus FcR on NK cells

**Fig 6:**
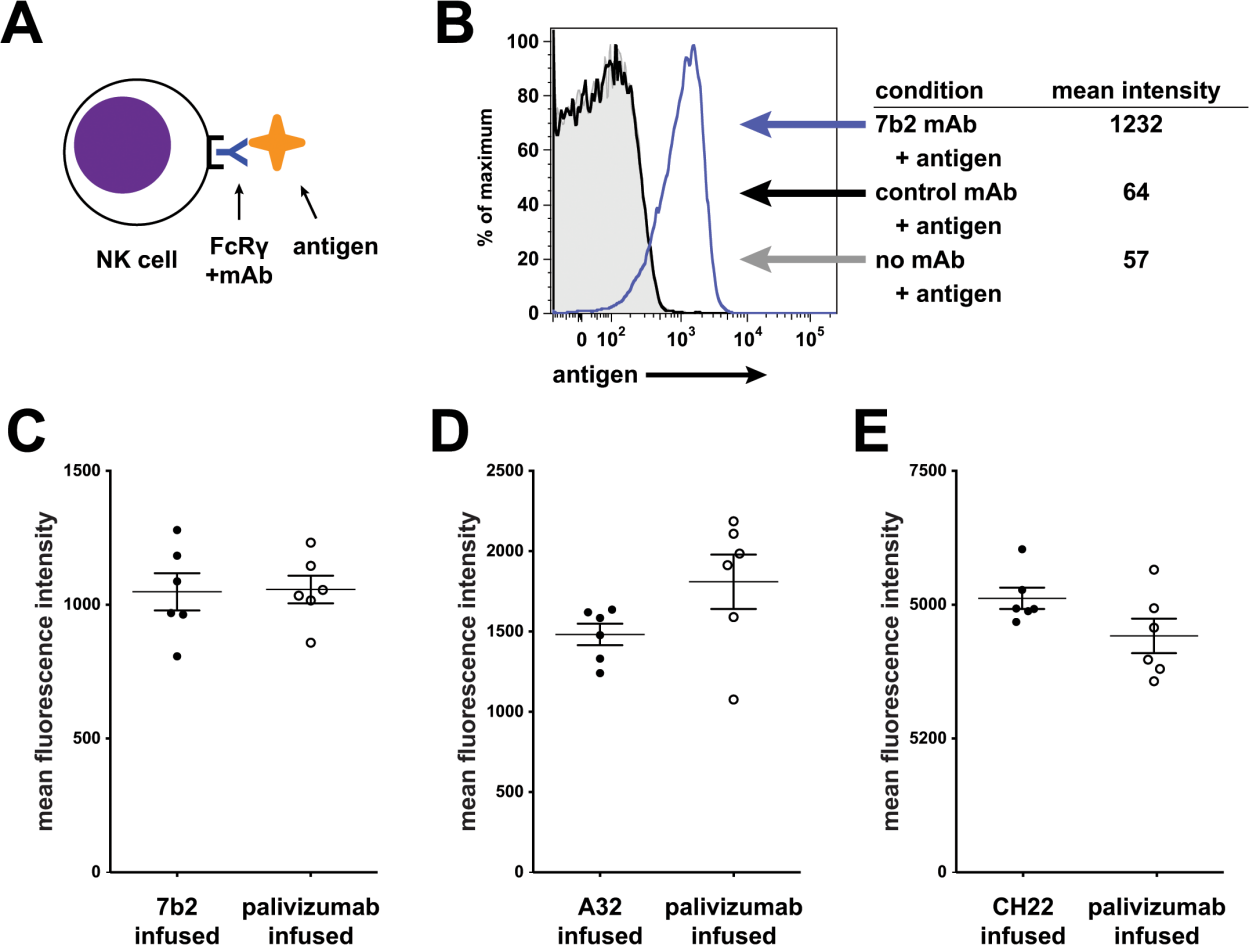
7B2 IgG1_AAA, A32 IgG1_AAA and CH22 IgG1_AAA mAb binding to rhesus FcR on NK cells. **(A**) Schematic of experiment: Rhesus PBMC were incubated with mAb and detected with fluorescently labeled antigens specific for the mAb being tested. (**B)** Rhesus PBMC gated on CD16+ NK cells were analyzed for binding of an HIV-1 gp41 immunodominant region reagent. Representative data: gray curve shows binding in the absence of mAb; the black curve shows binding to a control mAb. The blue curve shifted to the right shows binding of the reagent to 7B2 mAb bound to NK cells. (**C-E**) Assay of PBMC from animals infused in this study. Mean fluorescence intensity of rhesus NK cells for each animal is shown (grouped here by their actual grouping in the passive infusion study). In each case, PBMC were tested using lots of mAbs used for the infusion study. Antibody-reagent pairs are as follows: C. HIV-1 gp41 immunodominant region peptide tetramer with 7B2 mAb (**D**) HIV-1 gp120 A244 with A32 mAb (**E)** HIV-1 gp120 V3 loop peptide tetramer with CH22 mAb. No differences were found between groups for each infusion set.

To determine if the human HIV-1 specific mAbs can engage the rhesus FcR on NK cells, we examined binding of the mAbs to CD16 on rhesus NK cells by flow cytometry (Fig 6). Peripheral blood mononuclear cells (PBMCs) were isolated at a pre-infusion time point from each of the rhesus macaques enrolled in the passive infusion study (both palivizumab and HIV-1 mAb). Binding of 7B2 mAb, A32 mAb and CH22 mAb to these cells was measured to ensure that there was intact FcR-Ab engagement in these animals. In all rhesus macaques tested, there was substantial binding of the infused mAb to rhesus NK cells.

### Pharmacokinetic (PK studies)

**Fig 7:**
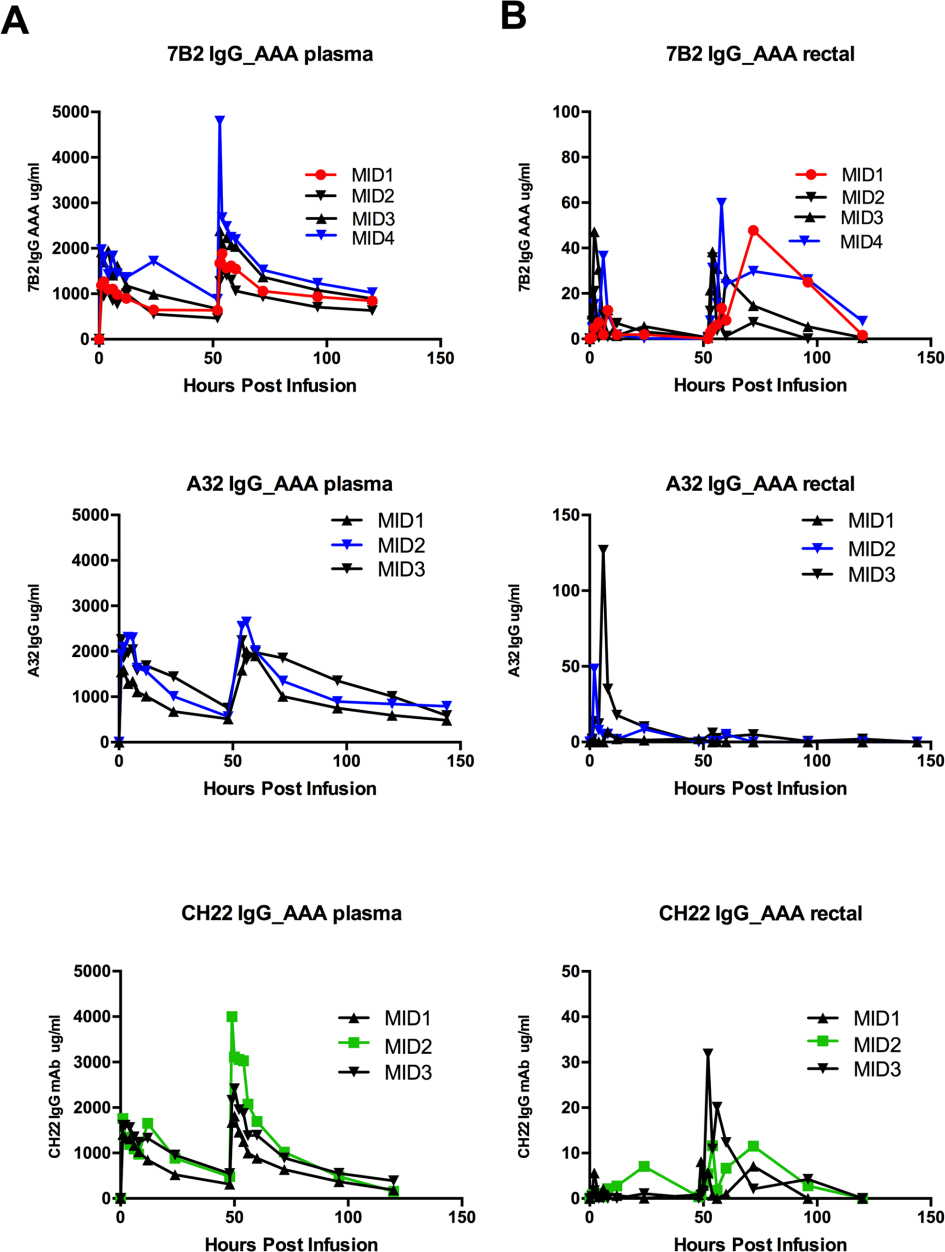
7B2 IgG1_AAA, A32 IgG1_AAA and CH22 IgG1_AAA mAb concentrations in (A) plasma and (B) rectal secretions. Concentrations of mAb were measured by a binding assay with the infused antibody as a control for calculating concentration equivalents of Ab binding to Env protein (μg/ml). Visible red blood cells in the rectal weck elutions were observed at time points post infusion for some animals.

*In vivo* PK studies were performed prior to passive protection studies for all antibodies to determine the concentrations and the half-lives of the antibodies in circulation and at the mucosal sites (Table 3; Fig 7). Two rhesus monkeys that were infused once with 7B2 IgG1_SEK IgG1 at 30 mg/kg had ~10 μg/ml of 7B2 IgG1_SEK in the rectal secretions. For the PK study using 7B2 IgG1_AAA IgG1, the Ab was administered to three rhesus monkeys twice at 50 mg/kg at 0 and 48 hours [67]. This resulted in a peak concentration of 30 μg/ml of 7B2 IgG1_AAA in the rectal secretions after the first infusion and ~90 μg/ml after the second infusion.

**Table 3:** mAb concentrations at time of challenge.

mAb PK Study	Mean Plasma μg/ml (range)	Time of Challenge	Mean Rectal μg/ml (range at time of challenge)	# Animals (Infusion Dose)
**CH22 IgG1_AAA**	1324.1 (887–1696)	60 hr (12 hrs post 2^nd^)	6.7 (1.0–12.3)	N = 3 (50 mg/kg)
**7B2 IgG1_AAA**	2035.4 (1484–2678)	54 hr (6 hrs post 2^nd^)	27.4 (4.3–38.3)	N = 4 (50 mg/kg)
**A32 IgG1_AAA**	1913.8 (1545–2258)	0 hr (post 1^st^)	0.96 (0.02–2)	N = 3 (50 mg/kg)

The average and range of antibody concentrations in plasma and rectal secretions from the PK study are indicated for the times that the subsequent infusion-challenge study was performed.

We also tested the non-neutralizing, A32 IgG1_AAA mAb that is one of the more potent of the ADCC-mediating antibodies, and is known to bind the surface of virus infected CD4^+^ T cells [18,20,65], but does not bind to Env on virions, and thus is unable to capture infectious virions. To address the question of whether the commercially prepared anti-RSV control antibody, palivizumab, may, in some way have affected transmission, we produced another control antibody, the influenza neutralizing anti-hemagglutinin IgG1 mAb CH65 [68], using the exact same protocol and methods as used in the production of 7B2 IgG1_ AAA and A32 IgG1_ AAA. Finally as a positive control, we produced CH22, an anti-V3 mAb derived from the RV144 HIV-1 vaccine efficacy trial [36] that neutralized SHIV-BaL *in vitro* in the TZM-bl neutralization assay at an IC_50_ of 1.9 μg/ml, in contrast to 7B2 IgG1_AAA and A32 IgG1_AAA that did not neutralize SHIV-BaL (>50 μg/ml). With these new reagents, we completed PK studies to determine the time of peak mAb concentration at the mucosal sites to perform the challenge studies.

### Challenge of 7B2 IgG1_AAA, A32 IgG1_AAA and CH22 IgG1_AAA treated rhesus monkeys with SHIV-BaL

**Fig 8:**
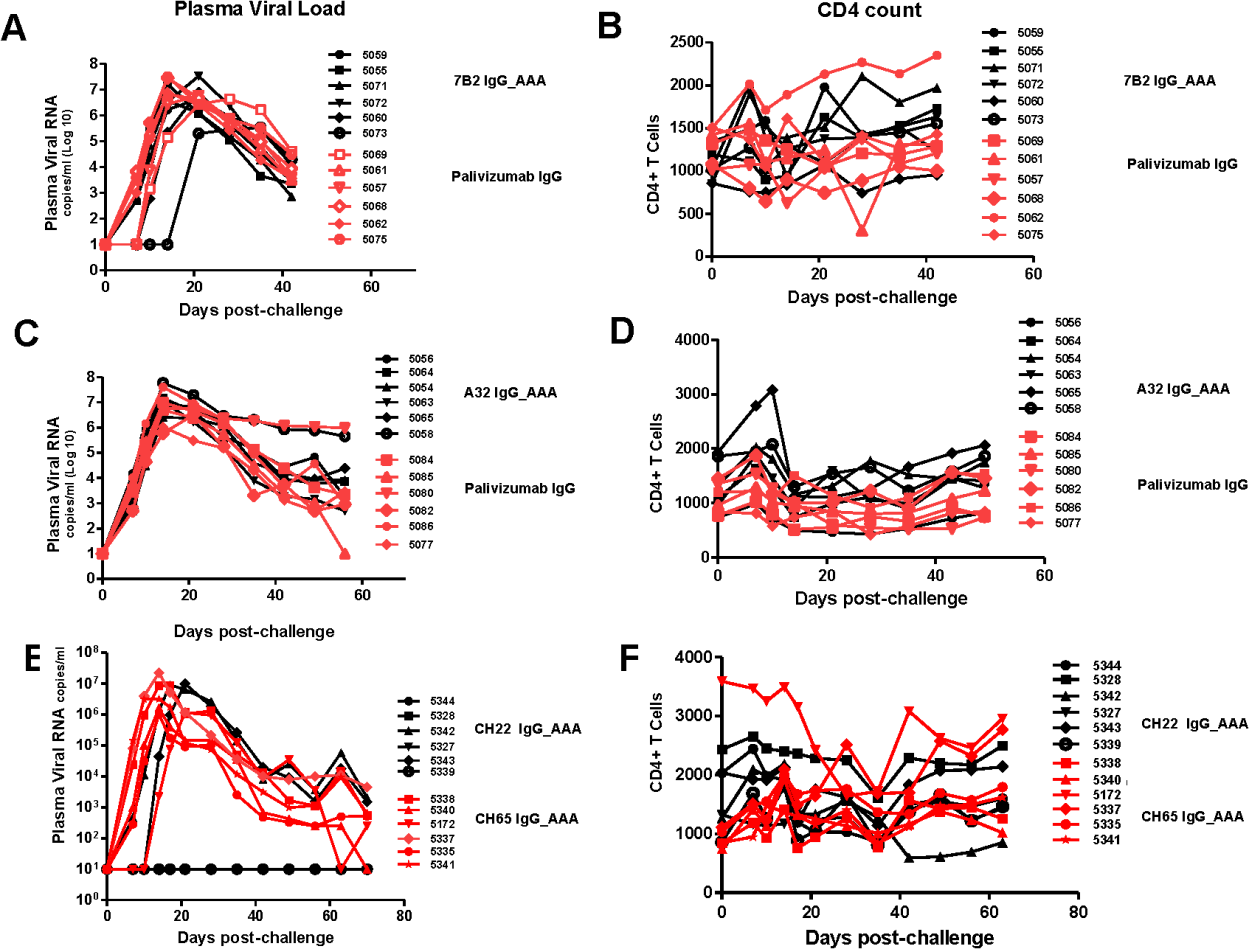
Viral loads and CD4 T cell counts following high dose SHIV BaL rectal challenge in rhesus macaques passively infused with 7B2 IgG_AAA, A32 IgG_AAA or CH22 IgG_AAA. **(A)** Plasma viral RNA levels and (**B)** CD4 T cell counts in 7B2 IgG_AAA and palivizumab IgG treated rhesus monkeys following challenge with SHIV-BaL. (**C**) Plasma viral RNA levels and (**D**) CD4 counts in A32 IgG_AAA mAb and control palivizumab IgG mAb passively infused rhesus monkeys following challenge with SHIV-BaL. (**E)** Plasma viral RNA levels and (**F)** CD4 Counts in rhesus monkeys following challenge with SHIV-BaL after passive administration of CH22 or CH65 IgG mAbs.

In the passive protection trial 7B2 IgG1_ AAA was administered at 50 mg/kg at 0 and 48 hours in six Indian-origin rhesus monkeys and the monkeys were challenged with 1 ml of SHIV-BaL (2×10^5^ TCID_50_/ml) via the intrarectal route at time 56 hours, the time of the peak antibody concentration post-second infusion. Another six rhesus monkeys received the control antibody palivizumab at the same dose and times as 7B2 IgG1_ AAA and were challenged at the same time based on the antibody concentration post-infusion in PK studies. We found that infusion of mAb 7B2 IgG1_AAA had no effect on peak viral load or on viral load at day 42 post-challenge (Fig 8A). Moreover, there was no significant impact on CD4 counts (Fig 8B). Thus, infusion of 7B2 IgG1_AAA resulting in peak mucosal antibody levels of 90 μg/ml at the time of challenge (Table 3, Fig 7), yet had no protective effect on SHIV-1 acquisition or control of viremia following high dose challenge with virus.

We then carried out studies using four new groups of Indian-origin rhesus macaques with 6 animals in each group, and each group infused with 50 mg/kg antibody at times 0 and 48 hours. The first group was infused with A32 IgG1_AAA ADCC antibody, the second group with the positive control CH22-IgG1_AAA V3 neutralizing antibody, the third group with negative control antibody, palivizumab, and the fourth group with new negative control anti-influenza neutralizing antibody, CH65. In order to insure the SHIV-BaL challenge coincided with peak plasma levels of mAb, monkeys infused with A32 IgG1_ AAA mAb and palivizumab control antibody were challenged intra-rectally with SHIV-BaL immediately following the first infusion whereas, monkeys infused with CH22 mAb and CH65 control mAbs were challenged at 60 hours using the same virus and same route. SHIV-BaL challenges were performed at the time of peak concentration of mAbs at the mucosal sites for each experimental mAb based on the findings from the PK studies.

Infusion of antibody A32 IgG1_ AAA into rhesus macaques had no effect on clinical acquisition of SHIV-BaL infection with 6 of 6 animals infected or on viral load or CD4 counts (Fig 8C and 8D). In contrast, infusion of the positive control V3-loop neutralizing antibody, CH22 IgG1 mAb, resulted in prevention of infection in 4 of 6 monkeys (Fig 8E). Similarly infusion of the negative control antibodies palivizumab and CH65 IgG1 (influenza neutralizing antibody) did not prevent infection in any of the monkeys (Fig 8C–8E).

### Enumeration of T/F viral genomes following high dose SHIV-BaL challenge

Keele and others have shown that in ~80% of cases of primary HIV-1 infection, one T/F viral genome (range 1–6) established productive clinical infection [33,69,70]. In men who have sex with men (MSM), one T/F genome (median 1, range 1–12) accounted for approximately 60% of cases, and in injection drug users this proportion fell to about 40% of cases (median number of T/F viruses 3, range 1–16) [34,71,72]. The *env* diversity present in the SHIV-BaL challenge stock (mean 0.3%, range 0–0.7%) is substantially less than the HIV-1 *env* diversity that is typically found in chronically infected humans (>>1%) [73], but it is nonetheless sufficient for distinguishing discrete T/F genomes (S3 Fig). We estimated the minimum number of T/F genomes responsible for productive SHIV-BaL infection (see Materials and Methods) in the 18 control animals to range from 1–27 with a median of 6.5 (Table 4). In contrast, after infusion of mAb 7B2 IgG1_AAA, the estimated numbers of T/F viruses was reduced 58% from the control Ab median of 6.0 to a median of 2.5 with a range of 1–5. Given that 60 sequences per animal were used to estimate numbers of T/F variants in the 7B2 IgG1_AAA treated and matched control animals, we could be 95% confident of sampling every variant that was present at >5% prevalence in each animal. Thus, the results revealed a significant reduction in the numbers of T/F viruses in the 7B2 treated animals compared with the six matched control animals (Mann-Whitney Rank Sum Test, p = 0.01) and when compared with the larger control group of 18 matched and unmatched control animals (p = 0.001) (Table 4).

**Table 4:** Number of Transmitted/Founder (T/F) viruses.

**7B2 IgG1_AAA^A^**	T/F variants	SGA Sequences per Animal	**A32 IgG1_AAA^B^ (Animal ID)**	T/F variants	SGA Sequences per Animal	**CH22 IgG1_AAA^C^**	T/F variants	SGA Sequences per Animal
5060	1	60	19–5054	2	34	5342	2	40
5073	1	60	18–5064	2	37	5343	2	42
5059	2	60	20–5063	3	42	5344	0	-
5071	3	60	22–5065	3	36	5328	0	-
5072	3	60	23–5058	5	37	5327	0	-
5055	5	60	16–5056	9	36	5339	0	-
**Median # T/F**	**2.5** *		**Median # T/F**	**3.0** *		**Median #T/F**	**0** *	
**Palivizumab IgG** ^A^ **(Control)**	T/F variants	SGA Sequences per Animal	**Palivizumab IgG** ^B^ **(Control)**	T/F variants	SGA Sequences per Animal	**CH65 IgG** ^C^ **(Control)**	T/F variants	SGA Sequences per Animal
5075	4	60	30–5086	6	34	5172	1	40
5062	5	60	28–5080	6	42	5335	8	41
5057	6	60	29–5082	6	37	5340	9	39
5069	6	60	24–5084	6	37	5337	11	40
5061	7	60	31–5077	9	42	5338	18	35
5068	12	60	26–5085	15	33	5341	27	45
**Median # T/F**	**6.0**		**Median # T/F**	**6.0**		**Median # T/F**	**10**	

^A^Minimum estimates of the number of T/F viruses for 7B2 IgG and palivizumab IgG treated rhesus macaques are shown. At a mean of 60 sequences per animal, there is 95% confidence that all variants that are >5% in prevalence have been enumerated.

*The difference in T/F variants between the 7B2 IgG_AAA treated animals and the negative control animals palivizumab IgG was statistically significant, p = 0.01; Mann-Whitney rank sum test, two tailed.

^B^ Minimum estimates of the number of T/F viruses for A32 IgG_AAA mAb and palivizumab IgG mAb treated rhesus macaques are shown. At a mean of 37 sequences per animal, there is 95% confidence that T/F variants with >8% prevalence in the population are represented.

*The difference in T/F variants between the A32 IgG_AAA treated animals and the negative control animals palivizumab IgG was statistically significant, p = 0.033; Mann-Whitney rank sum test, two tailed. ^C^ Minimum estimates of the number of T/F viruses for CH22 IgG_AAA mAb and CH65 IgG mAb treated rhesus macaques.

*The difference in T/F variants between the CH22 IgG_AAA treated animals and the negative control animals CH65 IgG was statistically significant, p = 0.011; Mann-Whitney rank sum test, two tailed.

**Fig 9:**
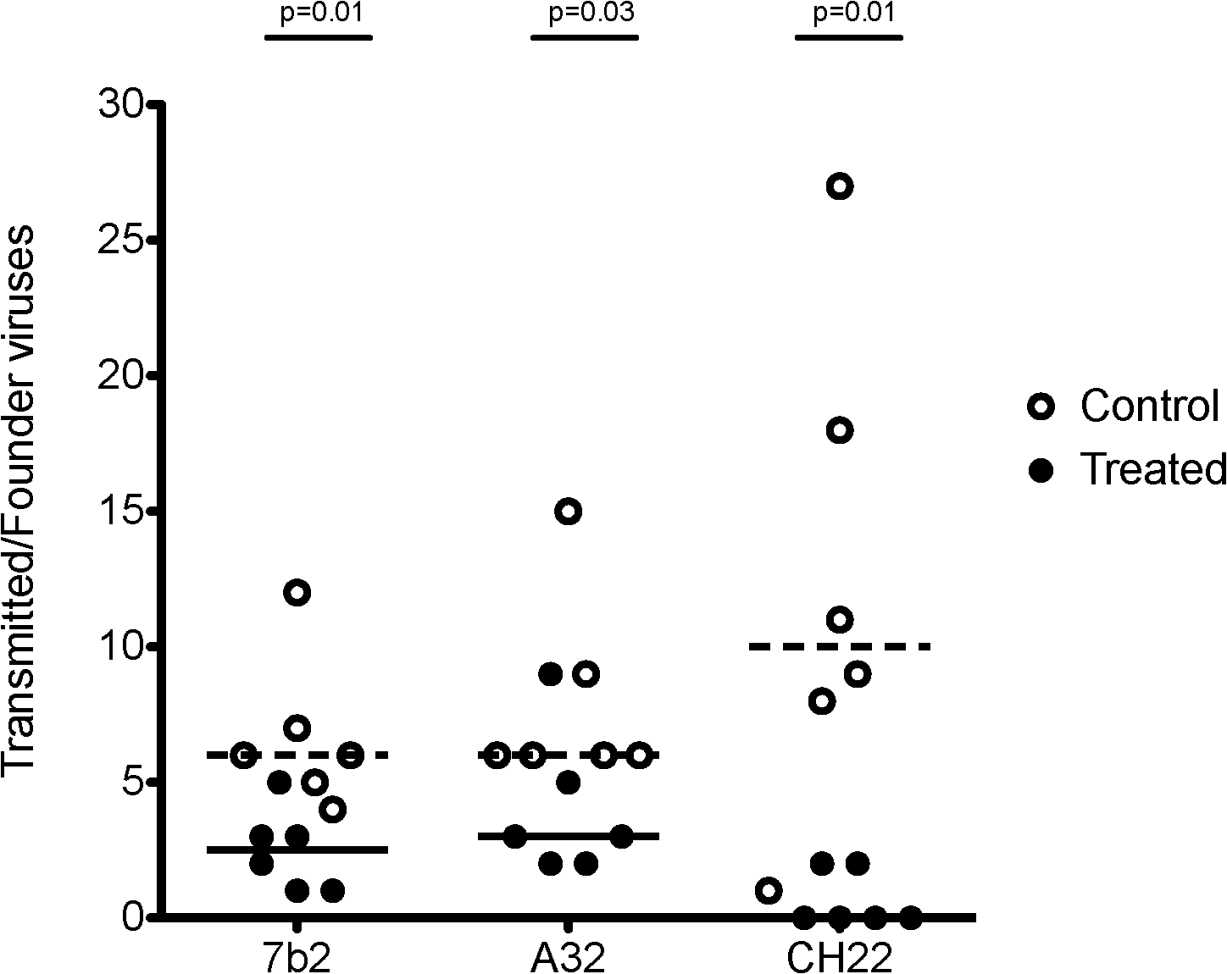
Minimum estimates of the numbers of Transmitted/Founder viruses resulting in productive clinical infection in rhesus monkeys following challenge with SHIV-BaL. Control animals (open circles) in the 7B2 and A32 studies were treated with palivizumab, which does not bind HIV BaL. Control animals in the CH22 study were treated with CH65 IgG_AAA, an anti-influenza antibody. Treated animals (closed circles) were infused with 7B2, A32, or CH22 monoclonal antibodies, as indicated. The dashed lines represent median numbers of T/F variants among controls, while the solid lines represent median numbers of T/F variants among treated animals. Listed p-values use the Mann-Whitney rank sum t-test to determine the significance of the differences in medians between treated and control animals in each group.

We next evaluated the number of T/F viruses in control mAb and A32 IgG1_AAA and CH22 infused monkeys (Table 4). The median number of T/F viruses in the first palivizumab control group performed with the 7B2 IgG1_AAA trial was 6, and the median number of T/F viruses in the second palivizumab control group was also 6. The median number of T/F viruses in the third control group infused with the new control antibody CH65 IgG1 was 10 with a range of 1 to 27 (Fig 9). Thus, among the 18 control animals, the median number of T/F variants was 6.5, and the difference in median T/F numbers between palivizumab and CH65 control antibody treated animals was not significant (p = 0.12, Mann-Whitney rank-sum test).

The median number of T/F viruses with the non-neutralizing ADCC-mediating A32 IgG1_AAA mAb was 3, a 50% reduction compared with the palivizumab control group where the median number of T/F viruses was 6 (p = 0.03, Mann-Whitney rank sum test) (Table 4). In the positive control CH22 antibody group, there were a median of 2 T/F viruses in each of the two infected animals, compared to a median of 10 founder viruses in the control group (p = 0.01 Mann-Whitney rank sum test) (Fig 9). Given a mean of 37 sequences per animal in the A32 IgG1_AAA study and a mean of 40 sequences per animal in the CH22 IgG1_AAA study, we are 95% confident that variants >8% prevalence in each animal are represented in the T/F enumeration. There was no evidence of selection pressure on the breakthrough viruses nor impact on the neutralization sensitivity of these viruses in animals treated with CH22 (S4 and S5 Figs). Additionally, for A32 IgG1_AAA and 7B2 IgG1_AAA passive infusion, there were no phylogenetically corrected signatures that were significant with a q value < = 0.3 that might suggest a selective pressure or sieve effect on breakthrough viruses. The lack of selection pressure on the antibody contact residues are shown in S6 Fig. The lack of evidence of virus escape from these mAbs is not surprising, since mutation in the highly conserved residues recognized by the A32 and 7B2 mAbs is likely destabilizing to the HIV-1 envelope glycoprotein and would alter virus fitness, and in the case of the A32 epitope, might decrease CD4 and co-receptor binding [74].

Thus, the high dose SHIV-BaL intra-rectal challenge model of rhesus macaque with an infection dose of ~10^5^ TCID_50_ was able to show protection by a V3 loop neutralizing antibody that neutralized the SHIV-BaL challenge stock. Using the same challenge model, both of the ADCC mediating antibodies 7B2 IgG1_ AAA and A32 IgG1_ AAA were not able to prevent productive clinical infection nor reduce viral load, but both mAbs were able to reduce the number of founder variants by ~50%. Importantly, none of the anti-HIV Env mAbs tested showed any evidence of enhancement of viral transmission.

## Discussion

In this study we have shown that two non-neutralizing antibodies, the gp41 targeted, ADCC-mediating, virus-capturing antibody 7B2 IgG1_AAA and the gp120 ADCC-mediating antibody A32 IgG1_AAA, each with mutations that enhance FcR binding [60], were able to limit the number of T/F viruses of SHIV-BaL in a high dose intra-rectal challenge model in rhesus macaques. While both antibodies did not reduce the frequency of clinical infection by SHIV-BaL, they did reduce the number of founder viruses by ~50%. In 7B2 IgG1_AAA treated animals, the median number of T/F viruses was reduced from 6 to 2.5 (p = 0.01). In A32 IgG1_AAA treated animals, the median number of T/F viruses was reduced from 6 to 3 (p = 0.03). In animals treated with the positive control V3 loop antibody CH22, virus transmission was eliminated altogether in 4 of 6 animals and the numbers of T/F viruses in the remaining two animals reduced to two compared with a median of 10 in the control group (p = 0.01).

There are caveats to the interpretation of these findings. First, the numbers of animals in treated and control groups were small (n = 6 for each). Second, the diversity in the SHIV-BaL challenge stock is limited (median *env* diversity 0.3%; range 0–0.7%), so distinguishing between discrete T/F genomes and genomes that acquire shared mutations post-transmission can be problematic. Third, the *in vivo* error rate of the SIV reverse transcriptase and the number of virus generations from infection to sampling must be estimated in distinguishing distinct T/F viruses from evolved viruses; we used previous empirical data and mathematical modeling of early viral replication dynamics [33,73] to estimate the frequency of mutations that might be expected in the first 10–21 days of infection to guide the identification of T/F genomes (see Methods). Fourth, APOBEC-mediated G-to-A hypermutation can confound T/F lineage analysis [33]; in the analysis presented we deleted G-to-A hypermutated sequences from our analyses [30,33,73]. Of note, the results regarding reduction in the number of transmitted strains in the presence of the non-neutralizing antibodies were supported when all G-to-A APOBEC motifs were removed from the analysis [33,75]. Fifth, our measurements of T/F genomes are point measurements that depend on depth of sampling for their sensitivity. They are not corrected for variants that might be present but not observed, and they are not expressed as point estimates with confidence limits. Alternative approaches to estimating numbers of T/F genomes that account for these limitations are in development (L. Blair, B. Korber, T. Bhattacharya).

SHIV-BaL is a CCR5-tropic tier 1 virus that is relatively easy to neutralize and is not a pathogenic SHIV. This SHIV was used to establish a sensitive model for non-neutralizing antibodies and to determine if any protection was present compared to previous studies using SHIV-BaL that have shown full protection with bnAb infusions [67]. While the challenge dose was high, it was the least amount of virus that caused infection of 100% of challenged animals (2.0 x10^5^ TCID_50_). Previous work has demonstrated that non-neutralizing antibodies administered locally or systemically were not as potent in protecting against SHIVs in rhesus macaques as antibodies that neutralize the challenge SHIVs. The present study sought to define the protective capacity of antibodies that are not able to neutralize HIV-1 in the TZM-bl assay but can mediate ADCC and other types of anti-HIV-1 immune effector functions. One caveat of this study is that the amount of virus present in the challenge stock, 2.9 x 10^9^ copies/ml (2.0 x 10^5^ TCID_50_) far exceeds the average amount of virus in semen of untreated HIV-1 infected individuals which is 0.426 x 10^4^ copies/ml (range 0.01–6.9 x 10^4^) [76]. If this seminal viral load reflects the amount of virus responsible for natural infection in the RV144 clinical trial, in which ADCC responses correlated with lower risk of infection in vaccinees with low anti-Env IgA responses, then we used a SHIV challenge dose that was 5 orders of magnitude greater than what may be responsible for natural HIV-1 infection. Therefore, it is possible that, in order to achieve infection in 100% of the control animals, we were considerably above the challenge threshold for the amount of virus that can be controlled by these non-neutralizing Abs. Thus, transmitted/founder virus enumeration was a more sensitive approach to study the impact of these non-neutralizing antibodies on transmission in this high dose challenge model.

A vaccine induced antibody response is unlikely to induce a single antibody specificity at the plasma concentrations present during the time of challenge (1,324–2,034 μg/ml); however, the mucosal antibody concentrations present in this study at the lower end of the range (0.96–27.4 μg/ml) are likely concentrations to be induced by vaccination. Whether or not the level of mucosal antibody concentrations present in individual animals in this study played a role in protection or reduction of founder viruses is unknown, but is worth further study to better understand the antibody concentrations needed for protection.

Antibodies that neutralize HIV-1 in conventional neutralization assays protect in passive protection trials. However, these neutralizing antibodies must have breadth and potency to be effective when passively administered. Administration of bnAbs clearly demonstrates the relevance of mAb breadth and ability to neutralize the few R5 SHIVs that are available for testing as challenge isolates. Hessell *et al*. have shown that the combination of conventional neutralization activity and IgG FcR-mediated activity such as ADCVI provides optimal protection in the setting of passive protection trials [32]. However, they have also demonstrated that nonfucosylated antibodies with better ADCC function were not better at protection[77]. Thus, additional studies are needed to determine whether the most likely attributes of protective IgG antibodies are to have conventional neutralizing activity with sufficient breadth to be clinically relevant and to potentially be able to mediate FcR dependent anti-HIV-1 activities. Moreover, additional passive infusion studies utilizing antibodies engineered to have optimal FcRn binding [78] to improve the antibody half-life can provide a way to examine the role of different non-broadly neutralizing antibody specificities in protection in a low dose mucosal challenge study.

MAb 7B2 IgG1_AAA and similar gp41 antibodies capture a subset of infectious virions in a CD4–dependent manner, mediate ADCC, and neutralize BaL in macrophage cultures. In contrast, mAb A32 IgG1_AAA does not capture virons, does not mediate macrophage neutralization, but is a very potent mediator of ADCC [18]. There is some controversy on the role of macrophages either as a virus reservoir or as part of the initial foci of infection after rectal or vaginal challenge. However, we broadly tested Fc-mediated activity of these mAbs, including macrophage neutralization and phagocytosis to characterize their potential effector function *in vivo*. That both of these antibodies (but not control antibodies) limited the number of founder SHIV-BaL viruses suggests that if viruses with traits like the tier 1 SHIV-BaL are indeed involved in HIV-1 transmission, then these common types of dominant gp41 and gp120 antibodies may play a role in protecting against HIV-1 transmission. Burton *et al*. found a suggestion of protection in 2 of 5 challenged animals using the gp41 non-neutralizing mAb F240, but the result was not statistically significant [30]. The authors counted T/F viruses in this study but there was no reduction in T/F viruses by F240 IgG mAb in infected animals [30]. Recently, in a live SIV vaccine (SIVmac239delta Nef) model, gp41 reactive antibodies, with properties similar to F240 mAb were shown to correlate with protection [79]. Also in the Burton study, the CD4 binding site non-neutralizing Ab b6 IgG mAb appeared to result in enhancement in the numbers of founder viruses compared with control animals [30]. This was not seen in the present study for 7b2 or A32 mAbs.

Others have demonstrated that the ability of an antibody to block virion transcytosis through epithelia is a predictor of protection both in the setting of subjects who are exposed and uninfected, and in the setting of vaccination [80]. The 7B2 IgG1_AAA antibody has recently been reported to block transcytosis *in vitro* [81], although in our mucosal explant models, neither A32 IgG1_AAA nor 7B2 IgG1_AAA blocked HIV-1 infection in the explant model *in vitro*.

It has previously been recognized that the 7B2 IgG1_AAA mAb can capture both infectious and non-infectious virions [11] and that the ability to capture virions does not correlate with ability of an antibody to neutralize HIV-1 [64]. MAb 7B2 IgG1_AAA most likely recognizes gp41 stumps from which gp120 has been shed [54] or some other non-native form of envelope on the virion. Since virions can contain a mixture of native and non-native Env forms [54–57], it was conceivable that an antibody like 7B2 IgG1_AAA could provide some measure of protection by binding to non-native Env forms on infectious virions. We do not know whether virus capture, ADCC, both or neither was involved in limiting founder viruses by 7B2 IgG1_AAA. However, A32 IgG1_AAA also limited founder viruses. Thus, we hypothesize that the effector mechanism was ADCC or a mechanism other than virus capture and retardation of virus transport across epithelia. One reason for this limited effect on prevention of founder virus number by 7B2 IgG1_AAA may be that although 7B2 IgG1_AAA does bind to infectious virions, it does not bind to all infectious virions (Fig 3). We recently demonstrated that although mAb 7B2 IgG1 cannot capture all infectious virus, the combination of two mAbs, 7B2 IgG1 and the V2 antibody CH58 IgG1 mAb, increased the capture of infectious virions [12].

It was of interest that our positive control, CH22 mAb, a V3 mAb isolated from an RV144 vaccinee [36] did neutralize SHIV-BaL (1.9 μg/mL IC_50/_10.4 μg/mL IC_80_), mediated ADCC (peak activity at 50 μg/mL), and protected 4 of 6 macaques from SHIV-BaL infection. V3 specific antibodies, in the context low specific Env IgA responses, were associated with a reduced risk of infection in the RV144 trial [82]. However, the infecting viruses in RV144 were Tier-2, while CH22 mAb only neutralized tier 1 viruses. Thus the protective capacity of CH22 mAb for the RV144 setting remains unclear. Moreover, in contrast to the protection of 4/6 animals by the linear V3 specific CH22 antibody, PGT121, a glycan V3 bnAb protected all of the animals [83]. The difference in the protective capacities of PG121 and CH22 are likely due to the differences in their epitope recognition and breadth/ potency for primary isolates (i.e. PGT121 recognizes glycans in the V3 region and is a broadly neutralizing antibody, unlike CH22 that is against a linear V3 and neutralizes only Tier 1 viruses).

We also show the co-crystal structure of gp41 immunodominant region mAb 7B2 Fab with its gp41 nominal epitope. The most prominent finding in the structure was the presence of a cysteine-cysteine loop with intact disulfide bond in the center of the gp41 Env contact region. Most recently, the BG505 SOSIP.664 trimer structure has showed partial structural detail of the pre-fusion gp41, in particular an ordered, disulfide-bonded immunodominant loop structure that was buried inside the glycoprotein complex [15]. This disulfide-bonded immunodominant loop structure of HIV-1 gp41 is involved in gp41-gp120 interactions [47,84–86] and is homologous to similar regions in other enveloped viruses and key structural elements in those virus infection mechanisms [48,86]. The extended conformation and hydrophobic nature of the residues at the tip of 7B2 CDR-H3 are reminiscent of the properties of gp41 membrane proximal broadly neutralizing antibodies, specifically that they tend to bear long, highly mutated CDR-H3s [1,87,88]. However, 7B2 was not polyreactive [88], does not have its epitope in the membrane proximal external region [89], and the CDR-H3 is not exceptionally long [1,87,90]. The data in this report demonstrate an exposed gp41 structure at amino acids 596–606 of Env that is exposed both on gp41 on HIV-1 virions and on virus-infected cells. This is the first structure of an antibody complex that shows binding at the functionally important disulfide bond in the chain reversal region of a retroviral entry protein. The cyclical loop motif has been demonstrated to be critical for function in other retroviruses, in particular to help constrain the local structure of the chain reversal region and to participate in formation of the hairpin structures during the fusion process.

Vaccines that induce immunodominant gp41 antibody responses, targeting the same epitope as 7B2 IgG1_AAA mAb, are in HIV-1 clinical trials [91]. It is important to note that these vaccines induce this antibody specificity in the context of a polyclonal antibody response. A recent non-efficacious HIV-1 vaccine trial (HVTN 505) [92] used a gp140 that had the C-C immunodominant loop region bound by 7B2 IgG1_AAA deleted, and thus could not induce 7B2 IgG1_AAA-like mAbs. For those vaccine regimens that do induce antibody specificities similar to 7B2 mAb [91] (Seaton *et al*., in preparation), our study can shed light on potential antiviral functions induced by HIV-1 vaccination. In our current study, non-neutralizing antibodies with defined specificities and functional characteristics were tested individually and some restriction in the number of founder viruses was observed. Future proof of concept studies in NHP can combine different Abs such as A32 IgG1_AAA (conformational C1), 7B2 IgG1_AAA (linear gp41) and CH58 IgG1_AAA (V2) [65] to determine whether a well-defined polyclonal mixture of antibodies can improve protection against mucosal infection.

## Materials and Methods

### Ethics statement

Rhesus macaques (*Macaca mulatta*) were housed at BIOQUAL, Inc. (Rockville, MD), in accordance with the standards of the American Association for Accreditation of Laboratory Animal Care. The protocol was approved by the BIOQUAL's Institutional Animal care and Use Committee under OLAW Assurance Number A-3086-01. Bioqual is IAAALAC accredited. This study was carried out in strict accordance with the recommendations in the Guide for the Care and Use of Laboratory Animals of the National Institutes of Health (NIH) and with the recommendations of the Weatherall report; “The use of non-human primates in research”. All procedures were performed under anesthesia using ketamine hydrochloride, and all efforts were made to minimize stress, improve housing conditions, and to provide enrichment opportunities (e.g., social housing when possible, objects to manipulate in cage, varied food supplements, foraging and task-oriented feeding methods, interaction with caregivers and research staff). Animals were euthanized by sodium pentobarbital injection in accordance with the recommendations of the panel on Euthanasia of the American Veterinary Medical Association.

Human peripheral blood mononuclear cells and peritoneal macrophages from HIV-1 negative individuals were collected with IRB approval by the Duke Medicine Institutional Review Board for Clinical Investigations (Protocol Pro00006526, Pro00000873, Pro00009459) and from healthy human subjects enrolled in the UC Irvine Normal Blood Donors Program (HS #2002–2430). All subjects were consented following 45 CFR 46 and written informed consent was obtained by all participants. No minors were recruited into this study. Additionally, monocytes were purified from blood packs purchased from the blood bank, with written informed consent from the donors. The approval to collect and store tissue was issued by the Imperial Tissue Bank with reference number Med_RS_11_014. Approval to use the tissue was given under Project number R11021. Approval for this project was granted by the Tissue management Committee at Imperial College Healthcare Trust in July 2011, and ethics thus conveyed through this process by MREC Wales, reference number 07/MRE09/54.

### Monoclonal antibodies

The 7B2 IgG monoclonal antibody was isolated from a HIV-1 chronically infected subject using Epstein-Barr (EB) virus B cell transformation and heterohybridoma production [35]. To produce recombinant 7B2 IgG1 antibody, the variable regions of immunoglubulin heavy and light chain (V_H_DJ_H_ and V_L_J_L_) genes of 7B2 IgG1 were isolated from the 7B2 IgG1 cell line by RT/PCR using the primers and methods as described [93], where the V_H_ and V_L_ gene rearrangements expressed by 7B2 IgG1 were determined by sequence analysis and annotated using the IMGT database.

For structural studies, after affinity capture the 7B2 Fab was further purified via gel filtration chromatography using a HiLoad 26/60 Superdex 200pg 26/60 column at 1 ml/min with a buffer of 10 mM Hepes pH 7.2, 50 mM NaCl, 0.02% NaN_3_. Peak fractions of Fab were pooled and exchanged into 50 mM Hepes pH 7.5 via five dilute/concentrate cycles in an UltraFree 4 ml 10K MWCO, then run over a cation exchange column (Mono S 5/50 GL) at 1 ml/min. At pH 7.5, the Fab passes through the column and excess light chain binds. The excess light chain is later eluted with 50 mM Hepes pH 7.5, 1 M NaCl. At lower pH values, the Fab binds the column and can be eluted with a gentle salt gradient, e.g. 0–15%. Peak fractions of Fab were pooled and exchanged into 10 mM Na Hepes pH 7.5, 50 mM NaCl, 0.02% NaN_3_ via four cycles of dilution-concentration and brought to a concentration >20 mg/ml for subsequent dilution.

Two forms of recombinant IgG1 heavy chain Fc were produced, one with the wild type IgG1 sequence termed as 7B2 IgG1_ SEK, and the other termed 7B2 IgG1_AAA. 7B2 IgG1_SEK contains Fc region aa of S298, E333 and K334 and 7B2 IgG1_AAA contains the Fc region aa of S298A as well as E333A and K334A, amino acid mutations previously reported to augment antibody ability to bind to FcRIIIa and to augment antibody ADCC activity [60]. The 7B2 IgG1_AAA mAb was expressed in CHO cells (Catalent, Somerset, NJ).

The A32 IgG1 monoclonal antibody was isolated from a chronically infected HIV-1 infected patient using Epstein-Barr virus B cell transformation and heterohybridoma production [94]. To produce recombinant A32 IgG1 antibody, the A32 IgG1 V_H_DJ_H_ and V_L_J_L_genes were isolated from the cloned A32 IgG1 cell line by RT/PCR as described [93]. The V_H_DJ_H_ and V_L_J_L_ gene rearrangements of A32 IgG1 were determined by sequence analysis. The recombinant A32 antibody was expressed in CHO cells (Catalent, Somerset, NJ) as IgG1_AAA to be optimized for binding to FcRIIIa [60]. Palivizumab, a commercial anti-respiratory syncytial virus antibody (Medimmune, Inc, Quakertown, PA), as well as recombinant CH65, an anti-influenza hemagglutinin mAb [68], were used as negative controls in the passive protection studies. Finally, CH22 neutralizing mAb reactive with the V3 region of HIV-1[36] was produced as a recombinant CH22 IgG1_AAA mAb in CHO cells (Catalent, Somerset, NJ). As a control for CH22, the influenza mAb CH65 [68] was produced as recombinant IgG1_AAA in CHO cells using the same technologies for 7B2_AAA, A32_AAA and CH22_AAA mAbs.

### Antibody binding

MAb binding 7B2 IgG_AAA to peptides and gp140 proteins was performed by ELISA, by binding antibody multiplex assays [26,95] and by surface plasmon resonance (SPR) assays [96,97]. Epitope mapping of mAbs was performed by peptide microarray microarray [37,98]. Gp41_596-606_ peptides with N-terminal biotin tags were commercially synthesized for SPR experiments. They were the wild type peptide and each residue substituted with Ala in turn. An additional gp41_596-606_ peptide was synthesized with an acetylated N-terminus and amidated C-terminus for crystallography. SP400 peptide (gp41_579-622_) was synthesized solely for SPR [26].

*Surface plasmon resonance (SPR)*. SPR binding assays were performed on a BIAcore 3000 (BIAcore Inc, Piscataway, NJ) instrument at 25°C and subsequent epitope mapping was carried out using a BIAcore 4000 instrument. Data analyses were performed using the BIAevaluation 4.1 software (BIAcore) as previously described [88]. Each residue of the 11mer WGCSGKLICTT (gp41_596-606_) was mutated to Ala and the peptides were made with N-terminal Biotin tag to facilitate their coupling to a BIAcore streptavidin chip. The biotinylated 11mer and its alanine-substituted variants were screened for binding by 7B2 (at 20 μg/ml). Binding responses of an irrelevant antibody Synagis was used to subtract out responses due to non-specific interactions.

For the gp41-binding experiments, both 7B2 and the negative control Synagis at a 50 μg/ml concentration were flowed at a 50 μl/min rate over SP400 tetramer (~4000 RU) and recombinant gp41 MN (~5700 RU) immobilized on a CM5 chip. The Gp41MN is a commercially available protein (Immunodiagnostics, Inc. product number 1091) and SP400 is a gp41-derived peptide with sequence RVLAVERYLRDQQLLGIWGCSGKLICTTAVPWNASWSNKSLNKI which was commercially synthesized (CPC Scientific) and tetramerized with streptavidin tags. For the experiments testing reducing conditions, biotinylated peptides were coupled to a BIAcore streptavidin chip. The peptides were initially screened for binding by 7B2 (at 10 μg/ml) at a flow rate of 20 μl/min with a PBS running buffer. Binding response to biotinylated SP62WT (a peptide containing gp41 MPER sequence) was used to subtract out non-specific interactions. The BIAcore 3000 was then primed with PBS, 20 mM DTT running buffer. The peptides were screened for 7B2 (at 10 μg/ml) at 20 μl/min with the PBS, 20mM DTT running buffer. Non-specific interactions were accounted for using the previously stated method.

### Crystallography

Peptides of sequence WGCSGKLICTT (gp41_596-606_) were commercially synthesized with acetylated N-termini and amidated C-termini in both reduced and disulfide-bonded forms (CPC Scientific). Lyophilized peptides were solubilized to 100 mg/ml in DMSO with no further dilution in aqueous solution prior to mixing with 7B2 Fab in a 1:3 Fab:peptide molar ratio. The complex was then diluted with 10 mM Na Hepes pH 7.5, 50 mM NaCl buffer to a total protein concentration of 12.5 mg/ml. Fab-peptide complexes were screened against various sparse matrix screens. Plates were incubated at 20°C. Crystals of the Fab with cyclical peptide were observed within two weeks in the Hampton Research PegRX screen condition H9, a solution of 5% 2-propanol, 0.1 M citric acid pH 3.5, 6% PEG 20K. Crystals were replicated in a fine matrix screen about the hit condition, the optimal of which was 4–6% 2-propanol, 0.1 M citric acid pH 3.5, 8% PEG 20K.

Crystals were cryoprotected with reservoir solution supplemented with 30% ethylene glycol immediately prior to cryocooling. Data were collected at SER-CAT BM at 1 Å wavelength. Data were reduced in HKL-2000 [99] in space group P2_1_ (P12_1_1). Matthews analysis suggested two Fab-peptide complexes of approximate molecular weight 49 kD each in the asymmetric unit corresponding to a solvent content of 66% and a Matthews coefficient of 3.6 [100]. The structure was phased by molecular replacement in PHENIX [101]. Source models were the light chain of humanized antibody CC49 [102] (86% identity to the light chain of 7B2) and the heavy chain of an antibody to neuropilin [103] (77% identity to the heavy chain Fab fragment of 7B2) with its CDR-H3 deleted. Conformations of the CDRs were rebuilt and the peptide antigen was constructed *de novo* as features of the electron density map improved with refinement. Rebuilding and real-space refinements were done in Coot [104] with reciprocal space refinements in PHENIX [105] and validations in MolProbity [106]. Non-crystallographic symmetry was employed throughout refinement. Coordinates and structure factors have been deposited into the Protein Data Bank (www.rcsb.org) with accession code 4YDV.

### mAb binding to rhesus FcR on NK cells

Antibodies tested for binding to FcR were the same lots as used for the passive infusion studies. Detection reagents were made as described [20,107]. All experiments included aliquots of the same human PBMC control to verify experimental consistency. Aliquots of rhesus PBMC were incubated with the appropriate mAb (7B2 IgG, A32 IgG, CH22 IgG, CH65 IgG [negative control], or mock) at 4°C for 30 min. For experiments using gp120 Env proteins for detection, cells were then blocked with an anti-CD4 mAb (clone SK3; Biolegend, San Diego, CA) at 4°C for 15 min. Cells were washed with 1x PBS + 1% BSA and stained at 4°C for 30 min with an NK cell panel consisting of CD3 PerCP-Cy5.5, CD4 PE-Cy7, CD14 PE, CD20 FITC, CD32 APC (BD Biosciences, San Jose, CA); CD16 BV570, CD64 APC-Cy7 (Biolegend, San Diego, CA); and CD8 PE-TexasRed (Invitrogen, Carlsbad, CA). The NK cell cocktail also contained the detection reagent matched to the FcR-bound mAb: 7b2 detected with gp41 immunodominant region peptide tetramer (sequence biotin-GGGKQLQARVLAVERYLKDQQLLGIWGCSGKLICTTAV); CH22 detected with gp120 clade B consensus V3 peptide tetramer (sequence biotin-GGGTRPNNNTRKSIHIGPGRAFYTTGEIIGDIRQAH); A32 detected with gp120 A244 Env tetramer. Cells were washed again and resuspended in 2% formaldehyde in PBS and stored at 4°C prior to acquisition on a BD LSRII flow cytometer (BD Biosciences, San Jose, CA). Data were analyzed in FlowJo (TreeStar, Ashland, OR). Rhesus NK cells were gated as negative for CD3 and CD14, CD20 dim/negative, CD8 bright [108] Mean fluorescence intensity of detection reagent binding was assessed for CD16^+^ NK cells only.

### Monocyte phagocytosis assay

Briefly, a 1ml packed suspension of pooled sheep red blood cells (SRBCs) were washed 3 times in 0.85% NaCl and spun at 3,000 rpm for 5 min. The SRBCs were then added to 1ml of a 0.5mg/ml gp41 immunodominant peptide (SP400: sequence biotin-GGGKQLQARVLAVERYLKDQQLLGIWGCSGKLICTTAV) antigen solution and 10 ml of chromium chloride solution (0.1 mg/ml). The coupling suspension was incubated at 30°C for 40 minutes in a shaking incubator (100 rpm) and then centrifuged for 10 minutes at 1000xg, 4°C. Coupled cells were then washed with 0.85% NaCl and centrifuged again for 10 minutes at 1000xg, 4°C. Coupled cells were resuspended as a 25% solution (v/v) in HBSS. The antigen coupling of sp400 to SRBCs was analyzed via flow cytometry. Antibodies 17b IgG mAb and 7B2 IgG_AAA (10 μg/ml) were incubated with 100 μl of a 2% solution (v/v in 0.85% NaCl) of both uncoupled and sp400 coupled SRBCs at room temperature for 30 minutes. Cells were washed twice with 3ml FACS Buffer (1x PBS + 1% BSA, pH 7.2) and centrifuged at 3000 rpm, 5 min to pellet cells. Supernatant was aspirated, and cells were gently re-suspended via agitation. Goat anti-human FITC secondary antibody (1:50 dilution in 1xPBS) was then added to the SRBC suspensions and incubated for 30 min at room temperature. Cells were again washed twice with 3ml FACS Buffer and then resuspended in 1ml FACS buffer.

Antibodies 7B2 IgG_AAA and palivizumab (both at 10 μg/ml) were incubated with gp41 immunodominant peptide coupled SRBCs as well as uncoupled (control) SRBCs for 45 minutes on ice. SRBCs were then washed 3 times with PBS and centrifuged at 3000rpm, 5 min. In 4ml tubes, 5x10^6^ coupled or sp400 coupled SRBCs were mixed with 1x10^6^ human or rhesus PBMCs in 1ml RPMI 1640. Cells were pelleted by centrifugation at 3000 rpm for 5 minutes and incubated as a pellet at 37°C for 30 min. After incubation, the pellet was resuspended in the 1ml media supernatant. Cytopreps were prepared and stained with Wrights stain.

### Surface plasmon resonance of MAb binding to FcRs

MAb binding to recombinant purified Fc receptors (FcRs) was performed using Surface Plasmon Resonance. Full sequences of the Fc receptors (recombinant FcRγI (CD64), FcRγIIa (CD32) and FCRγIIIb (CD16) (R&D Systems)).and the purification protocol of the rhesus FcRs is described elsewhere (Cocklin et al, 2014 *in preparation*). Briefly, rhesus FcR allelic variants were synthesized by Genscript with a hexa-histidine tag and cloned into the pcDNA3.3 mammalian expression vector (Invitrogen, Carlsabad, CA). Stable 293 HEK cell lines expressing rhesus FcR variants were prepared by nucleofection (AMAXA, Lonza) and antibiotic selection. Supernatants containing the soluble FcRs were harvested, centrifuged at 3500rpm for 1h and the supernatant was filtered through 0.45μm membranes prior to purification by Immobilized metal affinity chromatography (IMAC) using the Profinia protein purification system (Biorad, Hercules, CA). IMAC eluates were incubated with human pooled IgG sepharose for to remove serum protein contamination and for functional confirmation. IgG eluates were concentrated and dialysed into PBS, pH7.4. Purity was confirmed at >95% by SDS PAGE and coomassie staining and bicinchoninic acid (BCA) assay was used to determine the protein concentration.

### PBMC, monocyte/macrophage and TZM-bl neutralization assays

PBMC and HIV-1 envelope pseudotyped virus neutralization assays were performed as described [109,110]. Ability of the mAb to inhibit HIV-1 infection of monocyte/macrophages was performed as described [9]. Briefly, human peripheral blood (PB) monocytes were differentiated into macrophages and seeded on 48 well plates. Cells were infected with primary R5 HIV-1 BaL at a concentration of 200 ng/mL viral p24 antigen in the presence or absence of different concentrations of 7B2 IgG_AAA Ab and cultured in AIM plus Glutamax 1 and GM-CSF (10 ng/mL, R&D System). Productive infection was quantified by flow cytometry by detection of intracellular viral p24 antigen in MDM after 48 hours of culture. The percentage of infected cells in presence of Abs compared to control infected cells was determined. The TZM-bl neutralization assay was performed with the SHIV BaL challenge stock and the antibody concentration (μg/ml) at which relative luminescence units (RLUs) were reduced 50% compared to virus control wells were reported.

### Rectal explant culture and dissemination of infection by dendritic cells

Surgically resected specimens of intestinal tissue were collected at St Mary’s Hospital, London, United Kingdom, after receipt of signed informed consent. All patients were HIV negative. All tissues were collected under protocols approved by the Local Research Ethics Committee. Colorectal tissue was obtained from patients undergoing rectocele repair and colectomy for colorectal cancer. Only healthy tissue obtained 10 to 15 cm away from any tumor was employed. Following transport to the laboratory, muscle was stripped from the resected tissue, which was then cut into 2- to 3-mm3 explants comprising both epithelial and muscularis mucosae. Colorectal explants were maintained with Dulbecco’s minimal essential medium containing 10% fetal calf serum, 2 mM L-glutamine, 2.5 μg/ml Fungizone (Life Technologies) and antibiotics (100 U of penicillin/ml, 100 μg of streptomycin/ml, and 80 μg of gentamicin/ml) at 37°C under an atmosphere containing 5% CO2 as previously described [66]. Antibodies at 50 μg/ml were pre-incubated with cell free HIV-1_BaL_ (5 x 10^4^ TCID_50_) for 1 hour at 37°C. The 2-3mm^3^ dissected colorectal tissue explants were then exposed to virus and antibody for 2 hours at 37°C. Following viral incubation explants were washed three times with PBS and placed into 96-well tissue culture plates and cultured with fresh media containing antibody at the same concentration. The next day the tissue explants were transferred into a new 96-well tissue culture plate and washed twice with PBS. Tissue explants were subsequently cultured for 14 days in 200 μl of medium supplemented. On days 4, 7, 11 and 14 post-infection, 100 μl of supernatant was harvested and replaced with 100 μl of fresh media without further antibody exposure. To assess migratory cells present in the overnight culture of explanted tissue, cells were washed twice with PBS and co-cultured with 4 x 10^4^ PM-1 indicator T cells in 200 μl of medium containing antibodies at 50 μg. The supernatant was collected on days 4, 7, 11 and 14 post-infection and replaced with fresh media without further antibody exposure. All assays were performed in triplicate unless otherwise stated and controls included: virus only; medium only; and antibody isotype controls. Levels of p24 in tissue explant supernatants at day 11 post-infection were quantified using HIV-1 p24 enzyme-linked immunosorbent assays (ELISA; SAIC-Frederick, Inc., Frederick, MD) according to the manufacturer’s instructions. Each experiment was performed in triplicate, using tissues from different donors.

### Peritoneal macrophage infection assay

Replication competent HIV -1 _BAL_ was utilized to infect tissue macrophages and blood-derived macrophages. Peritoneal tissue macrophages were obtained under informed consent by IRB approval at Duke University. Virus input was normalized to RNA copies/mL. HIV replication was quantified by measuring the amount of luciferase in macrophage lysates at either day 4 or day 14 post-infection. HIV production was quantified by measuring luciferase in TZM-bl reporter cells infected with macrophage supernatants collected at regular intervals (4, 7, 11, and 14 days post infection).

### Infectious molecular clones (IMC)

HIV-1 reporter viruses (provided by Dr. John Kappes and Christina Ochsenbauer, University of Alabama) used were replication-competent infectious molecular clones (IMC) designed to encode the BaL (subtype B) *env* genes in *cis* within an isogenic backbone that also expresses the *Renilla* luciferase reporter gene and preserves all viral ORFs. The Env-IMC-LucR virus used was NL-LucR.T2A-BaL.ecto (IMC_BaL_) [111,112].

### Infection of CEM.NKR_CCR5_ cell line with HIV-1 IMC

IMCs were titrated in order to achieve maximum expression within 36 hours post-infection by detection of Luciferase activity and intra-cellular p24 expression. We infected 2x10^6^ cells with IMC_BAL_ by incubation with the appropriate TCID_50_/cell dose of IMC for 0.5 hour at 37°C and 5% CO_2_ in presence of DEAE-Dextran (7.5 μg/ml). The cells were subsequently resuspended at 0.5 x 10^6^/ml and cultured for 36 hours in complete medium containing 7.5 μg/ml DEAE-Dextran. Infection was monitored by measuring the frequency of cells expressing intracellular p24. The assays performed using the IMC-infected target cells were considered reliable if the percentage of viable p24^+^ target cells on assay day was ≥ 20%.

### Indirect surface immunofluorescence analysis of HIV-1-infected CD4+ T cells

Binding of mAbs to the surface of infected cells was performed as previously described [18]. Polyclonal activated CD4^+^-enriched T cells were obtained and infected by spinoculation (1200 *x g* for 2 hours; [113]) with NL-LucR.T2A-BaL.ecto (IMC_BaL_). Cells spinoculated in the absence of virus (mock-infected) were used as a negative infection control. Following 72 hours of infection in RPMI 1640 medium (Invitrogen, Carlsbad, CA), supplemented with 20% Fetal Bovine Serum (FBS) (Gemini Bio-Products, West Sacramento, CA) (R20) in presence of 20 U/ml rhIL-2 (Peprotech, Rocky Hill, NJ) (R20-IL2), CD4^+^-enriched T cells were washed in PBS, dispensed in 96-well V-bottom plates at 1x10^5^ viable cells/well, and stained with a vital dye (LIVE/DEAD Fixable Aqua Dead Cell Stain, Invitrogen) to exclude non-viable cells from subsequent analyses. The cells were then incubated at 4°C for 25 minutes with the primary Ab. After two washes, cells were stained with Phycoerythrin (PE)-conjugated goat anti-human IgG secondary (2ary) Ab (Southern Biotech, Birmingham, AL) for 2 hours at 37°C. Cells were subsequently washed 3 times and fixed with 1% formaldehyde PBS. The samples were acquired on a LSRII (BD Biosciences) within 24 hours. A minimum of 10,000 total singlet events was acquired for each test to identify live events. Data analysis was performed using FlowJo 9.6.4 software (TreeStar Inc., Ashland, OR).

### ADCC assay

Luciferase-based ADCC assays were performed as previously described luciferase-based assay [65,114]. The HIV-1 IMC_BAL_ infected CEM.NKR_CCR5_ cell line (NIH AIDS Research and Reference Reagent Repository) was used as target cells. The target cells were incubated in the presence of 4-fold serial concentrations of mAbs starting at 40 μg/ml. Purified CD3-CD16+ NK cells obtained from a HIV seronegative donor with the F/F Fc-gamma Receptor (FcRγ) IIIa phenotype were used as effector cells. The cells were isolated from the cryopreserved PBMCs by negative selection with magnetic beads (Miltenyi Biotec GmbH, Germany) after overnight resting. The NK cells were used as effector cells at an effector to target ratio of 5:1. The cells and mAbs were incubated in duplicate wells for 6 hours at 37°C in 5% CO_2_. The final read-out was the luminescence intensity generated by the presence of residual intact target cells that have not been lysed by the effector population in presence of ADCC-mediating mAb. The % of killing was calculated using the formula: % killing = (RLU of Target + Effector well)—(RLU of test well)/ (RLU of Target + Effector well) * 100. In this analysis, the RLU of the target plus effector wells represents spontaneous lysis in absence of any source of Ab. The humanized monoclonal antibody (IgG1_k_) directed to an epitope in the A antigenic site of the F protein of respiratory syncytial virus, (Palivizumab (MedImmune, LLC; Gaithersburg, MD) was purchased from the manufacturer and used as a control.

### HIV-1 virion capture assays

#### HIV-1 virion capture assay (p24 Gag, plate based)

For capture of p24/virions on 96 well plates, the ability of mAb 7B2 IgG_AAA to bind to HIV-1 virions was assayed using the methods as described [59]. Briefly, 96-well microtiter plates were coated with anti-human IgG (Sigma, St. Louis, MO) at 200 ng/100 μl per well in 50 mM bicarbonate buffer (pH 9.6) overnight at 4°C. Plates were washed three times with PBS-0.05% Tween-20, and blocked with 200 μl of PBS-2% BSA for one hour at room temperature and washed three times with 200 μl of PBS-0.05% Tween-20. Saturating amounts (4 μg/ml) of mAbs 7B2 IgG_AAA, A32 IgG_AAA, 2G12 or negative control mAb P3xG3 were added in triplicates and incubated in the presence and absence of soluble CD4 for 2 hours room temperature. The plates were washed again three times with 200ul of PBS-2% BSA and incubated for 2 h at 37°C with 100 μl of HIV-1 virion preparations of HIV-1 SF162.B, BG1168.B, 6535.B, 6846.B and CAP 45.C at approximately 5 x 10^5 TCID_50_ each. The plates were washed five times with 200 μl of PBS-2%BSA. The bound viral particles were disrupted by addition of 150 μl of NP40 solution. The amounts of virus captured by mAb were measured by p24 antigen capture ELISA using p24 antigen capture ELISA kit based on the protocol as recommended by the manufacture (DuPont/NEN Life Sciences, Boston, MA).

#### Infectious virion capture (protein G column)

Monoclonal IgG was mixed with 1x10^7^ RNA copies/ml NL-LucR.T2A viral stock at final concentration of 10 μg/ml in 300 μl. The IgG and virion IC mixture were prepared *in vitro* and absorbed by protein G MultiTrap 96-well plate as described in the measurement of endogenous IgG-HIV-1 virion IC above. The virions (viral RNA) in the flow-through or captured fraction were measured by HIV-1 *gag* real time RT-PCR. Briefly, after washing to remove non-specific bound material, 560 μl AVL lysis Buffer from QIAmp viral RNA mini kit (QIAGEN, Inc.) was added and centrifuged at 1500 x g for 3 minutes. The viral RNA in the lysis buffer was purified with QIAmp viral mini kit according to the manufacturer’s instructions. The viral RNA present in each fraction was measure by HIV-1 gag real time RT-PCR. Briefly, the one-step RT-PCR amplification reactions were performed in MicroAmp optical 96-well plate (Applied Biosystems, Foster City, CA) in a 25-μl reaction mixture containing 1x one-step Taqman RT-PCR master mix, 900 nM forward and reverse primers, 200 nM probe, and the template RNA in a final volume of 25 μl. Reverse transcription was performed at 48°C for 30 min followed by activation of TaqGold at 95°C for 10 min. Subsequently, 40 cycles of amplification were performed at 95°C for 15 s and 60°C for 1 min. The HIV broadly neutralizing antibody 2G12 mAb were utilized as positive controls. The 25 μl flow-through or input was used to infect TZM-bl cells. Infection was measured by a firefly luciferase assay at 48 hours post infection as described previously [115]. Briefly, 100 μl supernatant was removed and 100 μl Britelite (Perkin Elmer) was added to each well. After 2 minutes incubation, the 150 μl lysis was used to measure HIV-1 replication as expressed as relative luciferase units (RLUs). Each sample was run in triplicate. The percentage of viral particles in the flow-through or capture fraction was calculated as: = flow-through or capture RNA / (Flow-through+ capture) x 100%. The percentage of infectivity was calculated as: Flow–through infectivity/ input infectivity x 100%. The positive control 2G12 mAb gave virus capture of 30.1± 1.4% at 10 μg/ml. The negative control, palivizumab and virus only control were utilized to establish the positivity cutoff. The cutoffs for iVirion capture and rVirion capture were 15% and 10%, respectively, based on the mean of negative controls ± SEM.

### Infectious virus capture assay (vRNA plate based)

To measure the captured infectious IC, we adopted the Ig-virus capture assay described previously [26,64]. Briefly, Microplates (NUNC) were coated overnight at 4°C with mouse monoclonal anti-human IgG (Southern Biotech) at a concentration of 1 μg/ml diluted in PBS. After coating and washing, coated plates were blocked for 2 h with PBS supplemented with 5% Goat serum, 5% milk, 0.05% Tween. The indicated concentration of antibodies was mixed with the viral stock containing 5X 10^6^ viral RNA and then centrifuged 90 min at 2,000 rpm. Then the mixture was centrifuged at 21,000 x g for 45 min at 4°C to remove the virus free antibody [116] the pellet was resuspended in the same volume of PBS. 50 μl of the IC mixture was added to each coated well in triple wells for a 90 min incubation. Then, the wells were washed 4 times and the indicator cell line (M7-luc or TZM-bl) was added. HIV-1 replication was assessed on day 5 after infection for M7-luc and on day 3 for TZM-bl. The infection was measured by the firefly luciferase assay and was expressed as RLU.

### Identification and enumeration of T/F viral genomes

T/F viral sequences were obtained by single genome amplification (SGA) followed by direct amplicon sequencing by methods modified from Keele *et al*. [73] and published in Klein *et al*. [117]. Viral RNA was purified from the first or second virus positive plasma sample from each animal by the Qiagen QiaAmp viral RNA mini kit and subjected to cDNA synthesis using 1X reaction buffer, 0.5 mM of each deoxynucleoside triphosphate (dNTP), 5 mM DTT, 2 U/mL RNaseOUT, 10 U/mL of SuperScript III reverse transcription mix (Invitrogen), and 0.25 mM antisense primer SHIVBalEnvR1 5’- CTG TAA TAA ATC CCT TCC AGT CC -3’ located in the nef open reading frame (nt 9458–9480 in SIVsmm239). The resulting cDNA was end-point diluted in 96 well plates (Applied Biosystems, Inc.) and PCR amplified using Platinum *Taq* DNA polymerase High Fidelity (Invitrogen) so that ≤30% of reactions were positive in order to maximize the likelihood of amplification from a single genome. A second round of PCR amplification was conducted using 1 μl of the first round products as template. SHIVBalEnvR1 and SIVsm/macEnvF1 5’-CCT CCC CCT CCA GGA CTA GC-3’ (nt 6130–6146 in SIVsmm239 vpx) were used in the first round PCR amplification step, followed by a second round with primers envB5-in 5’- TTA GGC ATC TCC TAT GGC AGG AAG AAG -3’ (nt 5960–5983 in the HXB2 tat coding region) and BKSIVsm/macEnvR261 5’- ATG AGA CAT RTC TAT TGC CAA TTT GTA -3’ (nt 9413–9436 in SIVsmm239 nef). PCR was carried out using 1X buffer, 2 mM MgSO4, 0.2 mM of each dNTP, 0.2 μM of each primer, and 0.025 U/μL Platinum Taq High Fidelity polymerase (Invitrogen) in a 20-μL reaction. Round 1 amplification conditions were 1 cycle of 94°C for 2 minutes, 35 cycles of 94°C for 15 seconds, 58°C for 30 seconds, and 68°C for 4 minutes, followed by 1 cycle of 68°C for 10 minutes. Round 2 conditions were one cycle of 94°C for 2 minutes, 45 cycles of 94°C for 15 seconds, 58°C for 30 seconds, and 68°C for 4 minutes, followed by 1 cycle of 68°C for 10 minutes. Round 2 PCR amplicons were visualized by agarose gel electrophoresis and directly sequenced using an ABI3730xl genetic analyzer (Applied Biosystems). The final amplification product was ~3160 nucleotides in length exclusive of primer sequences and included all of *rev* and *env* gp160, and 336 nucleotides of *nef*. Partially overlapping sequences from each amplicon were assembled and edited using Sequencher (Gene Codes, Inc). Sequences with ≥2 double peaks indicating amplification from multiple templates were discarded. Sequences with one double peak were retained as this most likely represents a Taq polymerase error in an early round of PCR rather than multiple template amplification; such sequence ambiguities were read as the consensus nucleotide. Sequence alignments and phylogenetic trees were constructed using ClustalW and *Highlighter* plots were created using the tool at http://www.lanl.gov. Detailed descriptions of the mathematical models of early sequence evolution, star phylogeny determination, estimates of viral fitness, and power calculations for estimating the likelihood of detecting transmitted *envelope* variants with minor representation are provided elsewhere [118].

In order to compare T/F numbers accurately across different control and antibody-infused groups, we ensured that the number of sequences per group was consistent. In the 6 animals infused with 7b2 and the 6 matched controls, T/F numbers were determined by using 60 sequences per animal, for a total of 720 env sequences. We can be 95% confident of detecting all variants that are at greater than 5% prevalence in each animal. For the A32-treated and matched controls, a mean of 37 sequences were used from each group (range 33–42 over all 12 animals). For the CH22 study, 6 control animals had a mean of 40 sequences each, with a range of 35–45, while the 2 infected animals with CH22 infusion had 40 and 42 sequences, respectively. The A32 and CH22 studies were powered to detect any variants greater than 8% prevalence with 95% confidence. Therefore, all T/F numbers shown are a minimum estimate.

The following rules were followed in order to identify and enumerate T/F variants: In clusters of related sequences determined through visual analysis of phylogenetic trees [FigTree version 1.4 (http://tree.bio.ed.ac.uk/software/figtree/)], we allowed sequences with up to 3 mutations to be part of the same cluster from days 7–14 post-infection. At day 21 or 28 we allowed up to 4 changes in any one sequence. Variants exceeding these limits were identified as the progeny of distinct T/F genomes. Changes at positions predicted by the Hypermut algorithm 2.0 to be potential G-A hypermutation caused by APOBEC 3G/3F were reverted for analysis if there were ≤ 2. Sequences that had ≥ 3 potential APOBEC 3G/3F mutated positions were not considered for the T/F analyses (Hypermut, http://www.hiv.lanl.gov [119]). Sequence clusters of ≥ 2 sequences with ≥ 2 shared mutations were classified as distinct T/F variants. Sequences representing recombinants between two distinct T/F lineages were identified as previously described [33] and were excluded from the analyses. Sequences were deposited in Genbank under accession numbers KR608795—KR610312 (http://www.ncbi.nlm.nih.gov/Genbank/).

### Nonhuman primate passive infusion studies with SHIV-BaL challenge

*Mamu-A01*-negative Indian-origin rhesus monkeys were housed and maintained in an Association for Assessment and Accreditation of Laboratory Animal Care accredited institution in accordance with the principles of the National Institute of Health. All antibodies were produced in CHO cells (Catalent, Inc., Somerset, NJ) and were administered by the intravenous route in rhesus monkeys. Monkeys were challenged with 1 ml of SHIV-BaL (2 × 10^5^ TCID_50_) by the intra-rectal route at times specified in the “Results” section for each of the antibodies tested. The SHIV-BaL challenge stock used in the present study was uncloned. The original SHIV-BaL molecular clone was developed by Pal *et al*. [120], which was transfected into cells to derive a virus isolate, which in turn was serially passaged in 4 pig-tail macaques and then expanded in the PM-1 cell line. Our laboratory expanded this SHIV-BaL isolate further in human PBMCs and generated a large volume stock for rhesus challenge experiments. Mean genetic diversity of Env determined by single genome sequencing in this stock is 0.3%, with maximum diversity of 0.7%.

### Plasma viral RNA measurement

SIV plasma viral RNA measurements were performed at the Immunology Virology Quality Assessment (IVQA) Center Laboratory Shared Resource, Duke Human Vaccine Institute, Durham, NC. Plasma viral loads were assessed using a Qiagen QIAsymphony DSP Virus/Pathogen Midi Kit using the QIAsymphony SP platform and real-time PCR reaction carried out on the StepOnePlus (Applied Biosystems) instrument. Data from the real-time PCR reaction was analyzed using the StepOnePlus software. The sensitivity of this SIV viral load assay is 250 copies per ml.

### CD4+ T lymphocyte subset analyses

CD4+ T lymphocyte subsets were determined by multi-channel flow cytometry for CD3, CD4, CD8, CD28, CD95, CCR5 and CCR7. CD4+ T lymphocyte counts were calculated by multiplying the total lymphocyte count by the percentage of CD3+ CD4+ T cells. Briefly, 100 μl of EDTA-anticoagulated whole blood was stained with anti-CD3-A700 (clone SP34.2), anti-CD4-PerCP Cy5.5 (clone L200), anti-CD8-APC H7 (clone SK1), anti-CCR5-PE (clone 3A9), anti-CD95 APC (clone DX2) all from BD Biosciences, anti-CD28-PE CY7 (clone CD28.2; eBiosciences), and anti-CCR7-FITC (clone 150503; R&D Systems). Fixed cells were collected (30,000 events) on a LSRII instrument using FACSDiva software version 6.1.1 (BD Biosciences) and data were analyzed using FlowJo Software (TreeStar, Ashland, OR).

## Supporting Information

S1 Fig Representation of the 7B2 paratope. The 7B2 Fab paratope is colored by CDR as labeled. The left-hand panels show the paratope head-on and the right-hand panels show the Fab rotated 90 degrees. (A) The bulk of the gp41 immunodominant loop paratope resides on antibody heavy chain constituents, particularly CDR-H3. (B) The same views are shown with a graft of a complete, threaded CDR-L1 as described in the text. Also shown on the left-hand figure is a shaded circle indicating the approximate location of the 3D6 paratope in the vicinity of CDR-L3,-H2, and-H3[17].
(PNG)

S2 Fig Trans-infection of co-cultured T cells and ADCVI. 7B2 and A32 are inactive against infection of (A) monocyte- derived dendritic cells (MDC) or (B) DC-mediated trans-infection of co-cultured T cells. (C) mAb 7B2 mediates ADCVI against SHIV BaL and SHIV SF162. CEM.NKr-CCR5 cells were infected with SHIV for 72 hours, and 7B2 or palivizumab (negative control mAb) and macaque PBMC effector cells (E:T = 10:1) were added. Seven days later, virus yield was measured by p27 ELISA. Virus inhibition is relative to the virus yield obtained with palivizumab.
(PNG)

S3 Fig Neighbour-joining phylogenetic trees of single genome sequences (SGS) of SHIV BaL env gp160 and flanking *tat*, *rev*, and *nef* sequences (3170 nt) from representative animals in each study. Sequences are indicated by filled circles (cyan—anti-HIV treated animals; red–control animals). Unique T/F variants are indicated v1-v9 and represent minimum estimates (see methods). “*” indicates sequences with G-A hypermutations. “^” indicates recombinant sequences. (A) 7B2-AAA treated rhesus macaque 5071 was infected by 3 T/F variants. palivizumab treated animal 5057 was infected by 6 T/F variants. (B) Animal 5063 was treated with A32-AAA antibody and was infected by 3 T/F variants. Animal 5084 was treated with palivizumab control antibody and infected by 6 T/F variants. (C) CH22-AAA treated animal 5343 was infected by 2 T/F variants. 2. Animal 5340 was treated with control antibody CH65-AAA and infected by a minimum of 9 T/F variants. The scale bar beneath each figure represents one nucleotide mutation (0.0003 diversity).
(PNG)

S4 Fig No selection pressure on the challenge virus for breakthrough infection with CH22 IgG_AAA mAb passive infusion. Comparison of the Env amino acid sequences among three founder viruses from breakthrough SHIV BaL challenge are shown.
(PNG)

S5 Fig Neutralization susceptibility of breakthrough viruses in CH22 mAb infusion SHIV BaL challenge monkeys (IC_50_ μg/ml). Neutralization of the SHIV-BaL P4 challenge stock and the breakthrough viruses by CH22 mAb. Values are the antibody concentration at which relative luminescence units (RLUs) were reduced 50% compared to virus control wells (no test sample). Values in bold are positive for neutralization and red indicates values < 5.0 μg/ml IC_50_.
(PNG)

S6 Fig No Selection pressure evident at the antibody contact sites. Comparison of the Env amino acid sequences of the antibody contact sites among founder viruses from breakthrough SHIV_BaL challenge for (A) 7B2 or (B) A32 mAb passive infusion are shown. Contact residues for A32 mAb like mAbs were previously published [23]. Mobile Layer 1 contacts are indicated in turquoise: T52, L53, C54, S56, A58, K59, A60, H61, V68, W69, A70, T71, H72, A73, C74, V75, P76, T77, D78, P79, N80; and Mobile Layer 2 contacts are indicated in green: Q103, E106, D107, S110, Q114, Y217, T219, A221.
(PNG)

S1 Table sCD4 increases mAb 7B2 virion capture. Antibodies were tested for virion capture in the presence or absence of soluble CD4 in a p24 virion capture assay.
(PNG)
