# Supplementary figures and images for: Human Non-neutralizing HIV-1 Envelope Monoclonal Antibodies Limit the Number of Founder Viruses during SHIV Mucosal Infection in Rhesus Macaques
Source: PLoS Pathog. 2015 Aug 3;11(8):e1005042. doi: 10.1371/journal.ppat.1005042 (PMC4523205; doi:10.1371/journal.ppat.1005042)

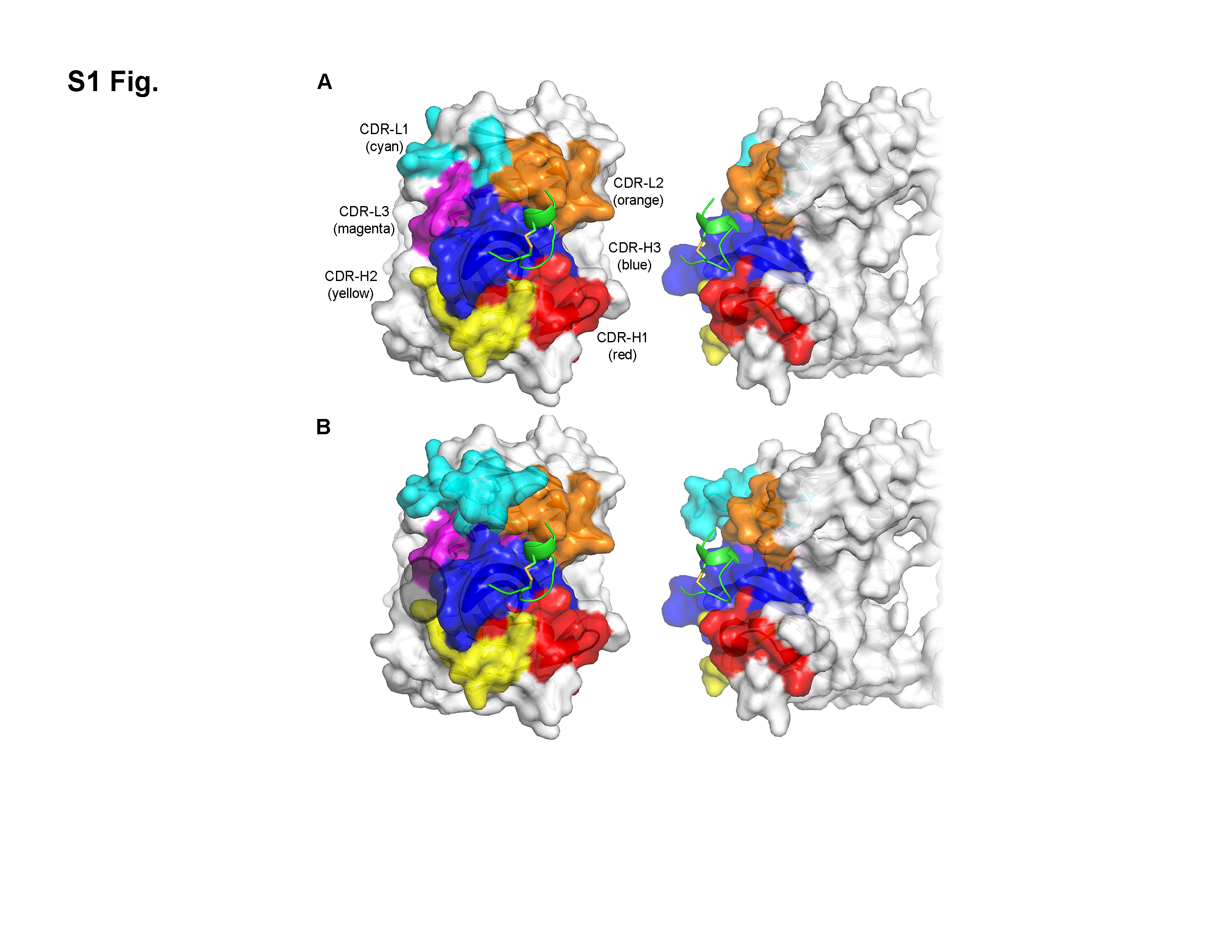

Supplement: S1 Fig — The 7B2 Fab paratope is colored by CDR as labeled. The left-hand panels show the paratope head-on and the right-hand panels show the Fab rotated 90 degrees. (A) The bulk of the gp41 immunodominant loop paratope resides on antibody heavy chain constituents, particularly CDR-H3. (B) The same views are shown with a graft of a complete, threaded CDR-L1 as described in the text. Also shown on the left-hand figure is a shaded circle indicating the approximate location of the 3D6 paratope in the vicinity of CDR-L3,-H2, and-H3[17]. (PNG) [file ppat.1005042.s001.png]

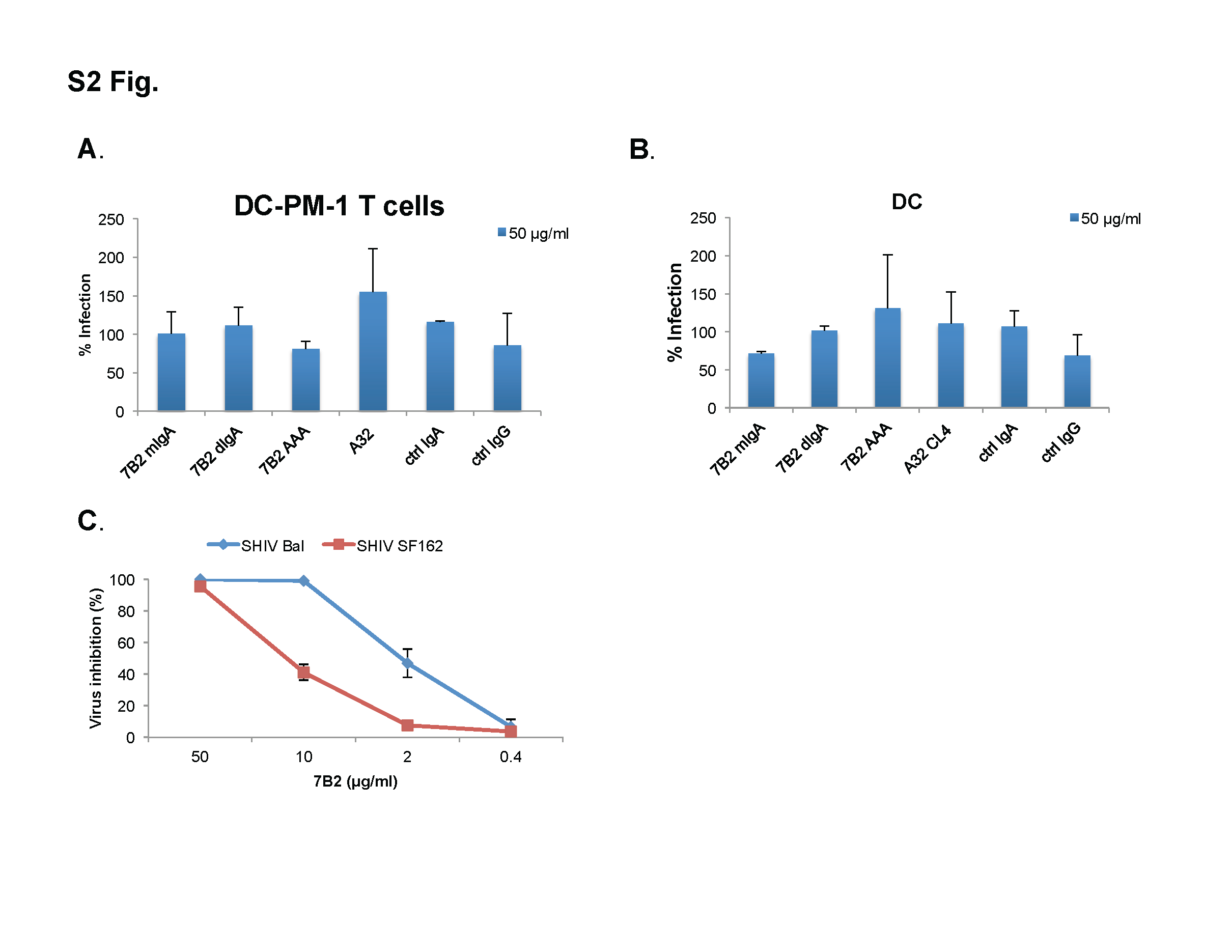

Supplement: S2 Fig — 7B2 and A32 are inactive against infection of (A) monocyte- derived dendritic cells (MDC) or (B) DC-mediated trans-infection of co-cultured T cells. (C) mAb 7B2 mediates ADCVI against SHIV BaL and SHIV SF162. CEM.NKr-CCR5 cells were infected with SHIV for 72 hours, and 7B2 or palivizumab (negative control mAb) and macaque PBMC effector cells (E:T = 10:1) were added. Seven days later, virus yield was measured by p27 ELISA. Virus inhibition is relative to the virus yield obtained with palivizumab. (PNG) [file ppat.1005042.s002.png]

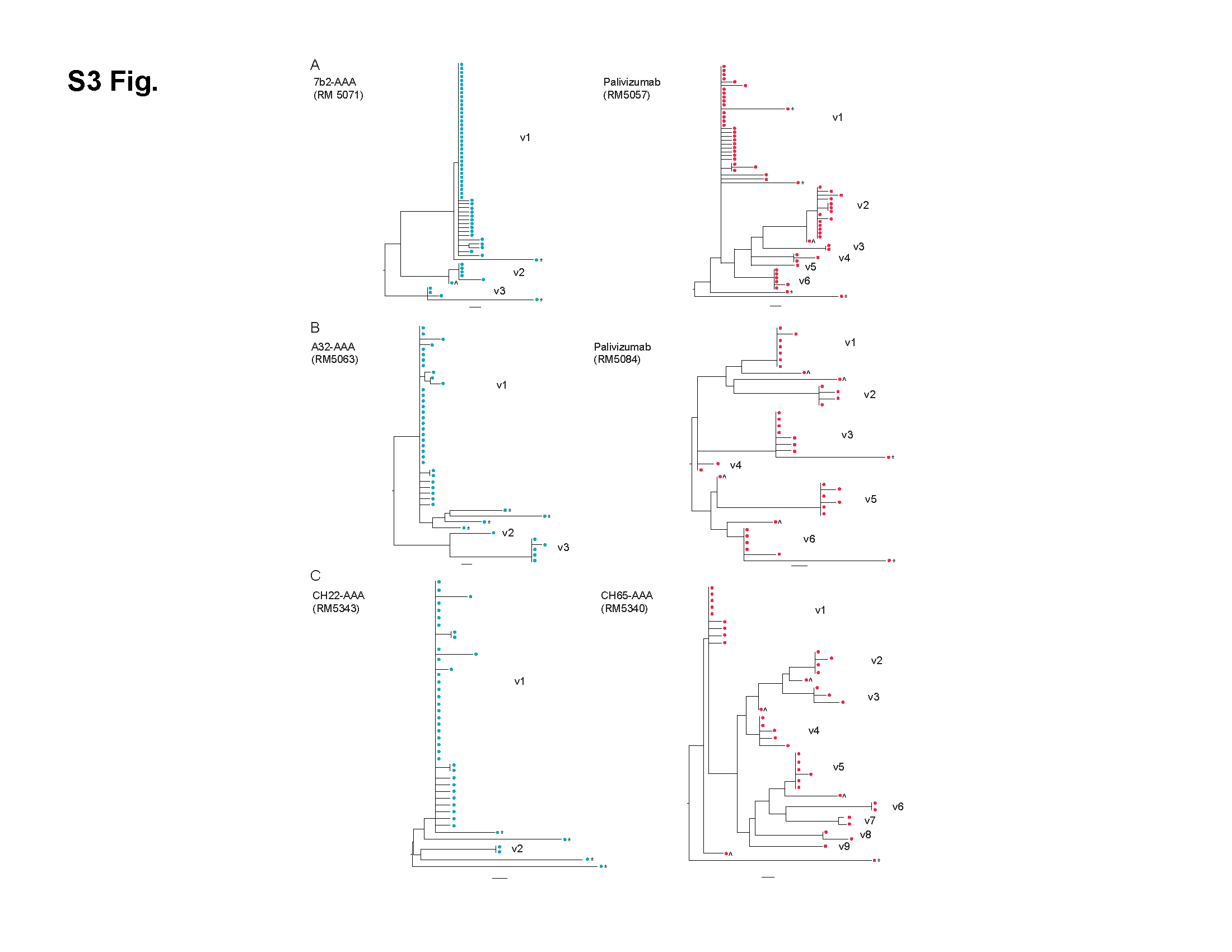

Supplement: S3 Fig — Sequences are indicated by filled circles (cyan—anti-HIV treated animals; red–control animals). Unique T/F variants are indicated v1-v9 and represent minimum estimates (see methods). “*” indicates sequences with G-A hypermutations. “^” indicates recombinant sequences. (A) 7B2-AAA treated rhesus macaque 5071 was infected by 3 T/F variants. palivizumab treated animal 5057 was infected by 6 T/F variants. (B) Animal 5063 was treated with A32-AAA antibody and was infected by 3 T/F variants. Animal 5084 was treated with palivizumab control antibody and infected by 6 T/F variants. (C) CH22-AAA treated animal 5343 was infected by 2 T/F variants. 2. Animal 5340 was treated with control antibody CH65-AAA and infected by a minimum of 9 T/F variants. The scale bar beneath each figure represents one nucleotide mutation (0.0003 diversity). (PNG) [file ppat.1005042.s003.png]

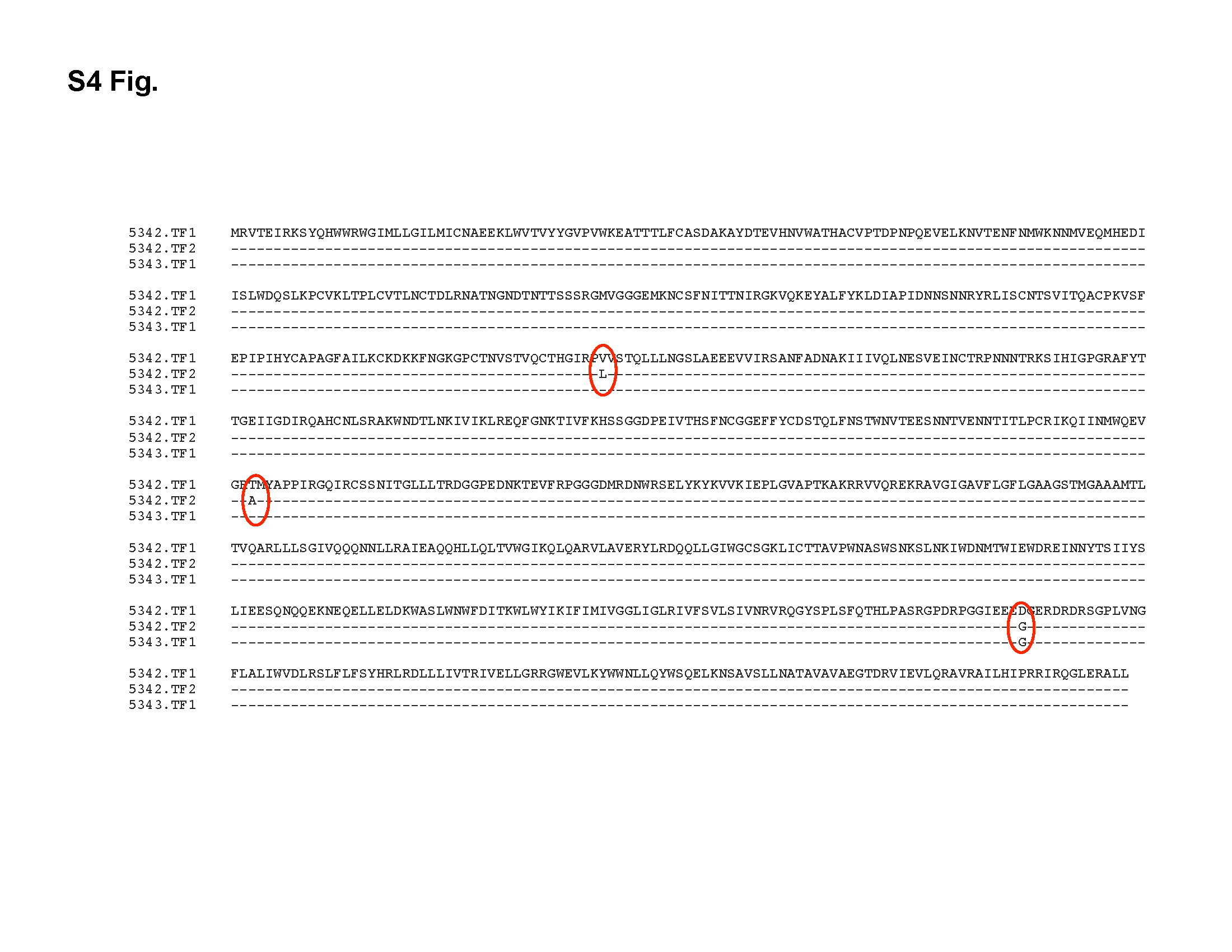

Supplement: S4 Fig — Comparison of the Env amino acid sequences among three founder viruses from breakthrough SHIV BaL challenge are shown. (PNG) [file ppat.1005042.s004.png]

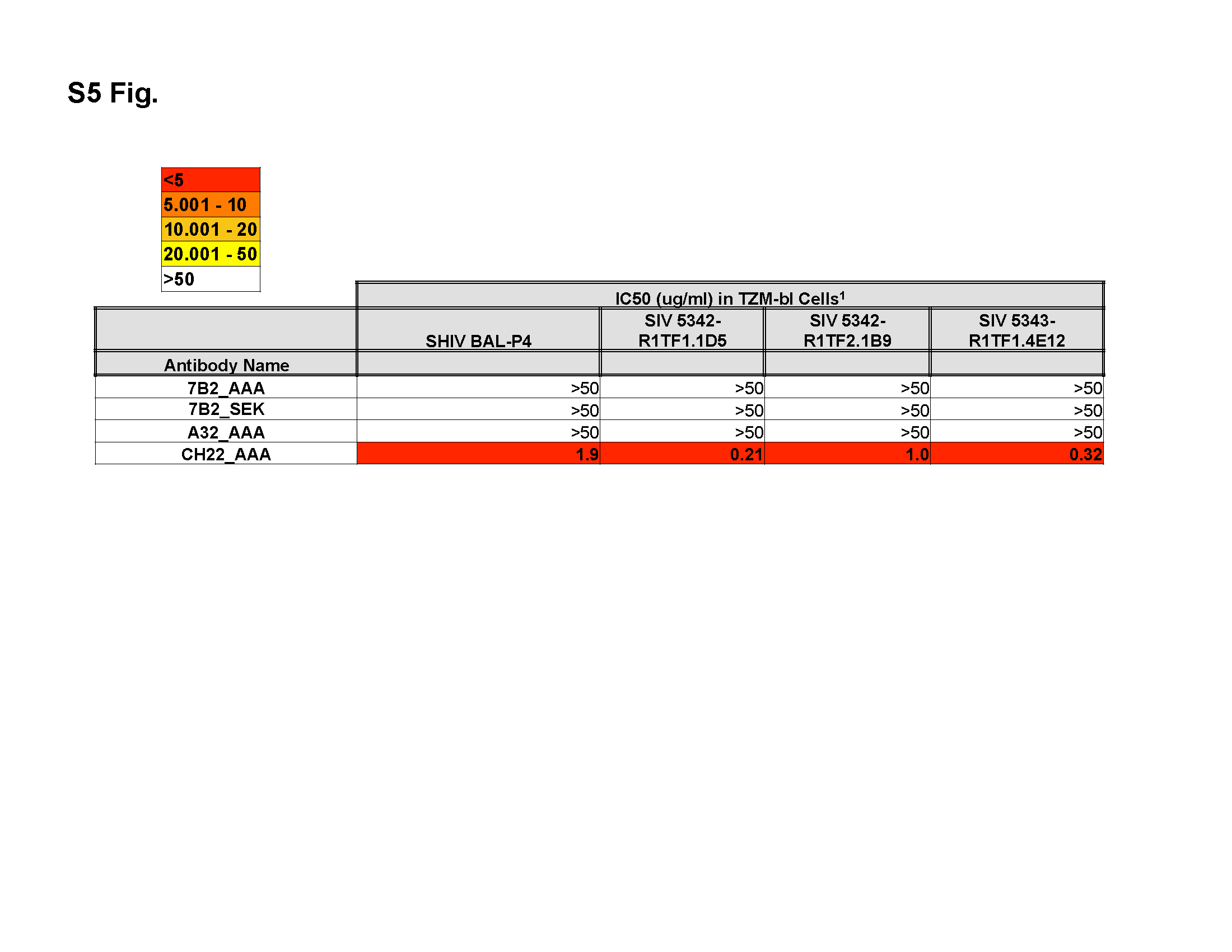

Supplement: S5 Fig — Neutralization of the SHIV-BaL P4 challenge stock and the breakthrough viruses by CH22 mAb. Values are the antibody concentration at which relative luminescence units (RLUs) were reduced 50% compared to virus control wells (no test sample). Values in bold are positive for neutralization and red indicates values < 5.0 μg/ml IC50. (PNG) [file ppat.1005042.s005.png]

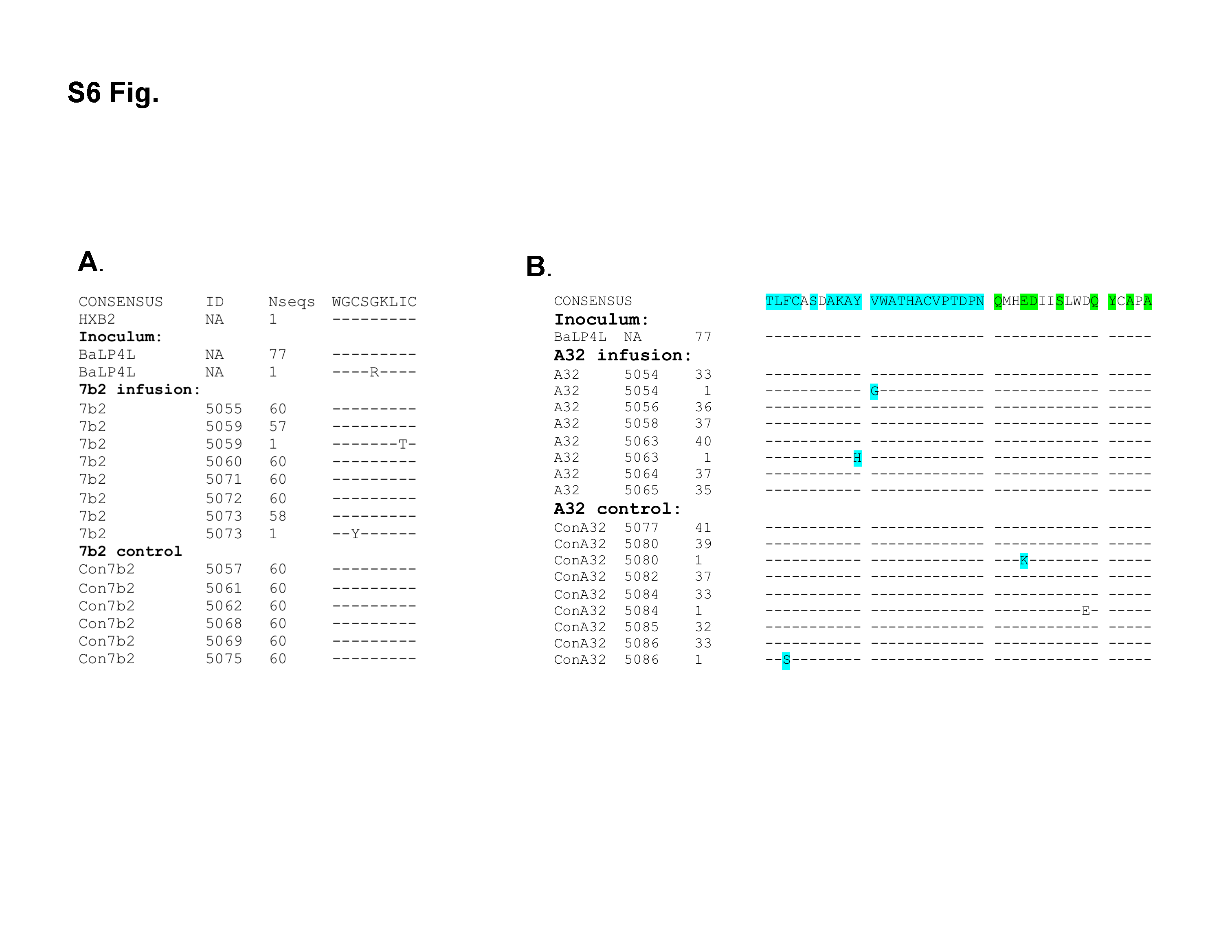

Supplement: S6 Fig — Comparison of the Env amino acid sequences of the antibody contact sites among founder viruses from breakthrough SHIV_BaL challenge for (A) 7B2 or (B) A32 mAb passive infusion are shown. Contact residues for A32 mAb like mAbs were previously published [23]. Mobile Layer 1 contacts are indicated in turquoise: T52, L53, C54, S56, A58, K59, A60, H61, V68, W69, A70, T71, H72, A73, C74, V75, P76, T77, D78, P79, N80; and Mobile Layer 2 contacts are indicated in green: Q103, E106, D107, S110, Q114, Y217, T219, A221. (PNG) [file ppat.1005042.s006.png]

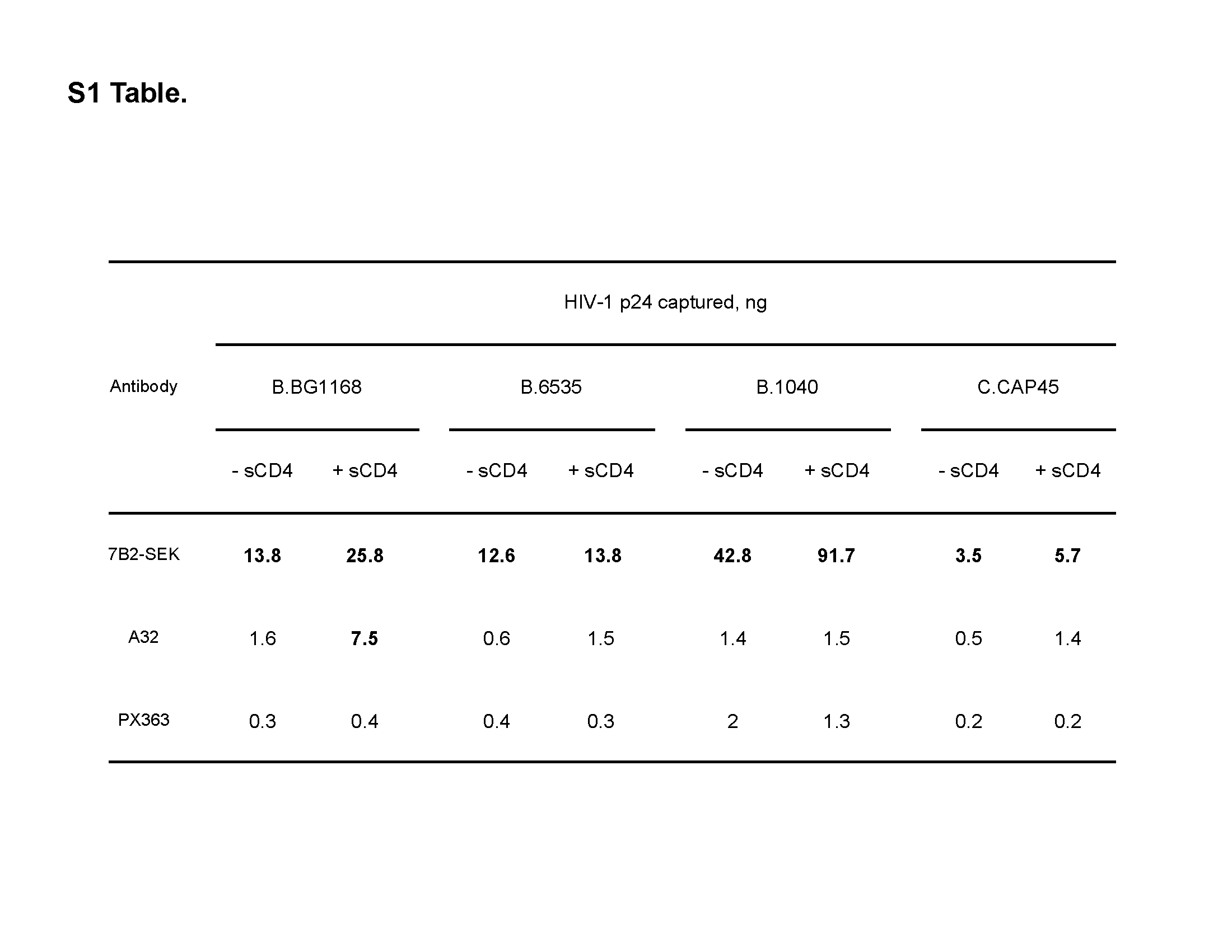

Supplement: S1 Table — Antibodies were tested for virion capture in the presence or absence of soluble CD4 in a p24 virion capture assay. (PNG) [file ppat.1005042.s007.png]
